# Enantioselective synthesis of tertiary α-chloro esters by non-covalent catalysis

**DOI:** 10.1016/j.tetlet.2015.01.124

**Published:** 2015-06-03

**Authors:** Richard Y. Liu, Masayuki Wasa, Eric N. Jacobsen

**Affiliations:** a Department of Chemistry & Chemical Biology, Harvard University, Cambridge, Massachusetts 02138, United States

**Keywords:** Asymmetric catalysis, Organocatalysis, Hydrogen bonding, Chlorination, Non-covalent interations

## Abstract

We report an enantioselective approach to tertiary α-chloro esters through the reaction of silyl ketene acetals and *N*-chlorosuccinimide. The reaction is promoted by a chiral squaramide catalyst, which is proposed to engage both reagents exclusively through non-covalent interactions. Application of the tertiary chloride products in stereospecific substitution reactions is demonstrated.

The development of new methods for enantioselective α-chlorination is motivated primarily by the utility of α-chloro carbonyl compounds as synthetic intermediates.^1,2^ While construction of α-secondary α-chloro stereocenters has been explored extensively using strategies such as enamine catalysis,^3^ few successful methods have been reported for enantioselective catalytic α-chlorination at tertiary centers.^4–7^ In the context of our studies of enantioselective reactions promoted through non-covalent catalysis,^8^ we envisioned that electrophilic chlorination of α-tertiary silyl enolates might be promoted by a chiral hydrogen bond donor with ancillary functionality capable of positioning the reacting partners in a specific orientation (Scheme 1).

The chlorination of cyclic silyl ketene acetal **1a** with *N*-chlorosuccinimide (NCS) was investigated as a model reaction (Table 1).^10^ Among the various subclasses of chiral dual hydrogen bond donors that have been developed over the past decade, tert-leucine-arylpyrrolidine derivatives with the general structure in **3**–**5** have proven remarkably effective in a wide range of enantioselective catalytic reactions. Whereas promising results were observed with thiourea catalyst **3**, substantially higher reactivity and enantioselectivity were obtained with the analogous urea and squaramide derivatives **4** and **5a** (entries 1–3). A systematic evaluation of arylpyrrolidino-sqaramide catalysts revealed a strong dependence of reaction ee on the expanse and orientation of the aromatic component.^11^ The 9-phenanthryl derivative afforded highest enantioselectivities, with smaller (e.g. 2-naphthyl, entry 7) and larger (e.g. pyrenyl, entry 8) substituents affording substantially poorer results.

Catalytic chlorination reactions with squaramide **5a** were found to be quite sensitive to reaction conditions (Table 2). Reactions carried out in dichloromethane (DCM)) afforded racemic product (entry 1), as NCS is fully soluble in that medium and the chlorination reaction appears to occur entirely through an uncatalyzed pathway. In contrast, NCS is only sparingly insoluble in toluene and ethereal solvents, and measurable levels of enantioselectivity are obtained in those solvents (entries 2–4). This suggests that one role of the squaramide catalyst may be to solubilize NCS, presumably through hydrogen binding interactions. Based on this hypothesis, we evaluated the addition of hexanes as an additive to further reduce solubility of NCS and thereby suppress the background reaction. Optimal results were obtained at −30 °C with the introduction of 10% hexanes (entries 5 vs 7). Further increase in proportion of hexanes had a deleterious effect on both yield and enantioselectivity, most likely due to the insolubility of catalyst **5a** in these solvent mixtures (entries 8 and 9).

The optimized enantioselective chlorination protocol was applied to several silyl ketene acetals derived from 2-arylbutyrolactones (Table 3). High ee’s were observed with substrates bearing neutral or slightly electron-withdrawing substituents on the aryl group (**1a–1e**, **1g**), but electron-donating substituents underwent chlorination with significantly poorer enantiocontrol (e.g., **1f**, **1h**). Low enantioselectivities were also observed with other classes of silyl enolate substrates (see Supporting Information). The product ee is slightly sensitive to the identity of the silyl group (substrates **1a** vs **1i** vs **1j**, Table 3), suggesting that the silyl group is still associated to the substrate in the enantiodetermining transition structure.^12^

α-Halocarbonyl compounds are excellent substrates for S_N_2 pathways,^13^ so we explored the possibility of effecting stereospecific substitution reactions at the tertiary α-position of product **2a** (Scheme 2). Treatment of **2a** with sodium azide cleanly effected the desired substitution reaction in nearly quantitative yield and with high stereospecificity. Likewise, substitution with phenylthiolate was accomplished at elevated temperatures, in moderate yield and similar stereospecificity (66% yield, 86% ee). Finally, reaction of **2a** with cesium fluoride and crown ether successfully yielded tertiary α-fluoride **8**, albeit in moderate yield (25% yield, 90% ee). Together, these represent two-step asymmetric net C–N, C–S, and C–F bond forming reactions of α-tertiary silyl enolates.

Previous studies of uncatalyzed reactions of silyl enol ethers with NCS have pointed to rate-determining formation of an ionic intermediate by Cl^+^ transfer from NCS to the silyl enolate.^9^ In the enantioselective, squaramide-catalyzed reaction described here, we propose that the polarized transition state leading to the ion pair intermediate may be stabilized by hydrogen bonding to the succinimide, with simultaneous stabilization of the developing positive charge on silyl ketene acetal through a cation-π interaction with the arylpyrrolidine (Scheme 3). The profound effect of the arene substituent size and orientation on reaction enantioselectivity (Table 1) is consistent with the crucial role of such an attractive interaction in defining the transition structure geometry. An alternative mechanism involving formation and reaction of a squaramide-bound enolate is unlikely given the observed dependence of ee on identity of the silyl group (see above).

In summary, we have developed a method for the enantioselective α-chlorination of α-tertiary silyl ketene acetals with NCS. The reaction is promoted by a chiral arylpyrrolidino squaramide catalyst that binds and activates the reactants through a network of non-covalent interactions. Synthetic application of these products in stereospecific substitution at the tertiary carbon was demonstrated. Identification and mechanistic characterization of related types of enantioselective catalytic ion-pairing pathways is the subject of ongoing work in our laboratory.

## Supplementary Material

SI

## Figures and Tables

**Scheme 1:**
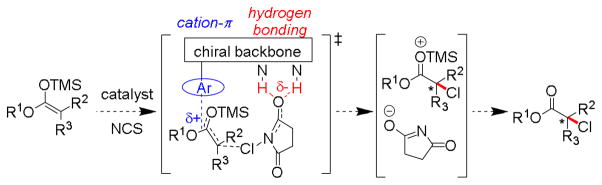
Catalytic Strategy for α-Chlorination Reaction

**Scheme 2:**
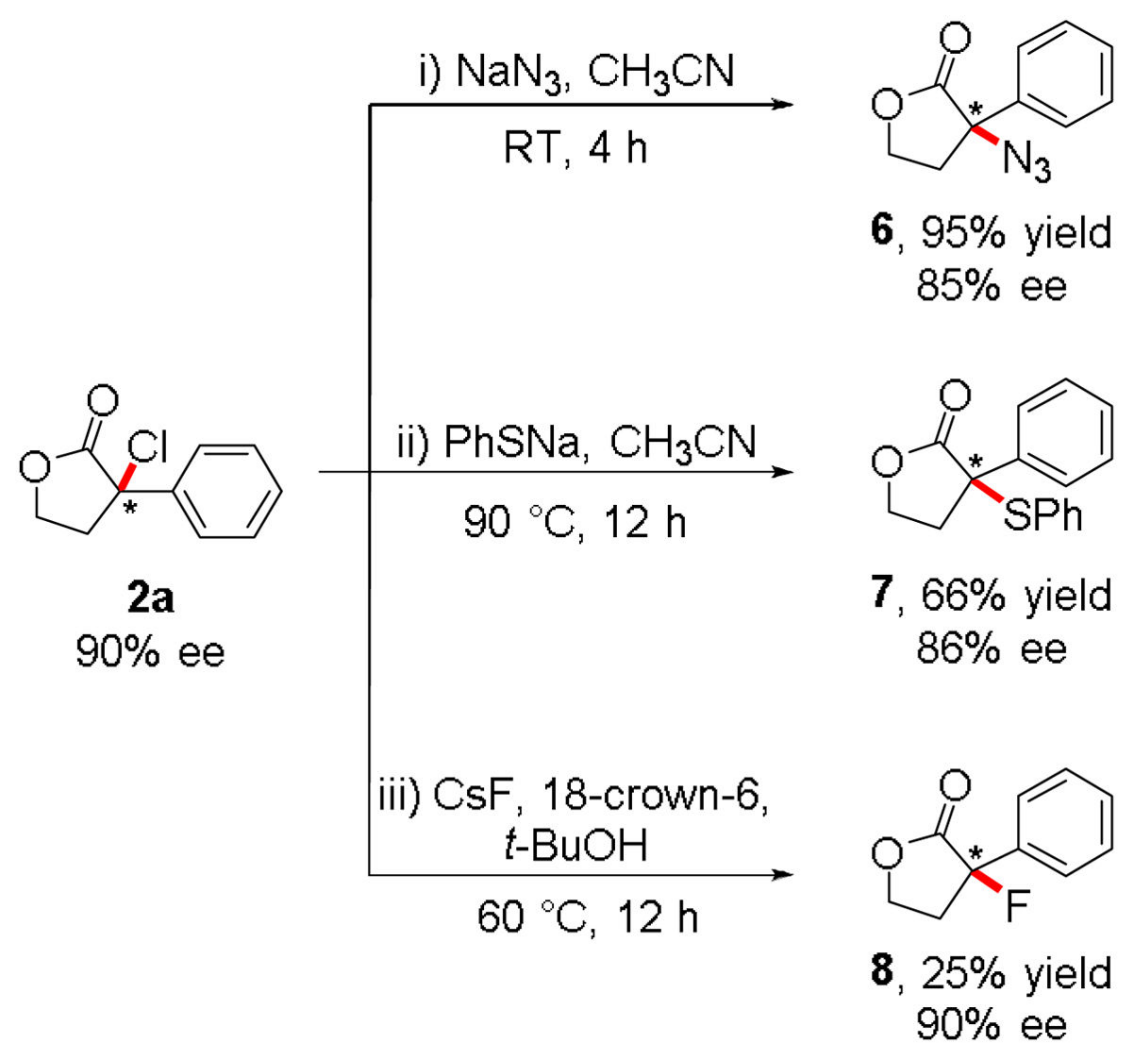
Substitution Reactions of **2a** *^a,b,c^* *^a^* Conditions: i) **2a** (0.5 mmol), sodium azide (1.0 mmol) in acetonitrile (4 mL) at room temperature for 4 hours. ii) **2a** (0.5 mmol), thiophenol sodium salt (1.0 mmol) in acetonitrile (0.5 mL) at 90 °C for 12 hours. iii) **2a** (0.1 mmol), cesium fluoride (0.3 mmol), 18-crown-6 (0.1 mmol) in tert-butanol at 60 °C for 12 hours. *^b^* Isolated yield of purified product. *^c^* Enantiomeric excess determined by HPLC analysis on commercial chiral columns.

**Scheme 3:**
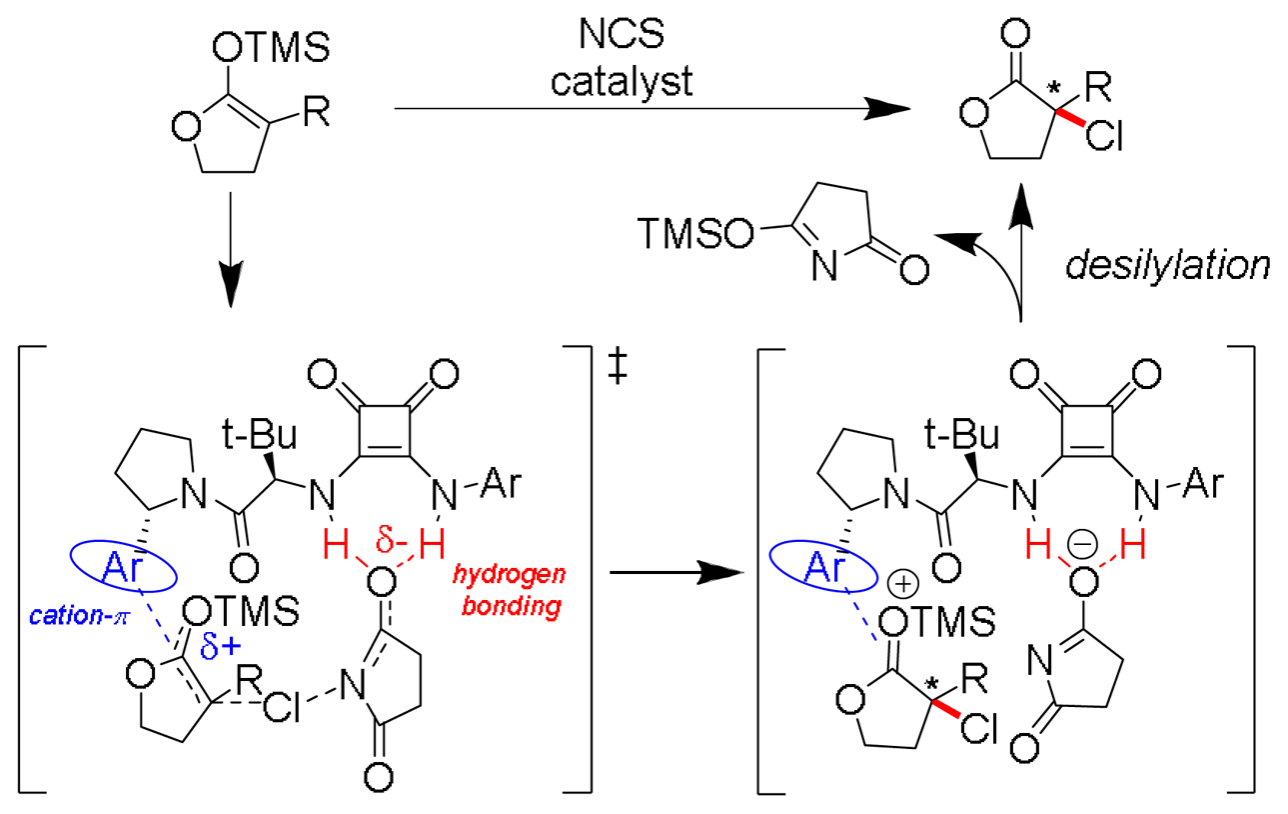
Proposed Mechanism of Chlorination Reaction

**Table 1:** Evaluation of Catalyst Structure^a^

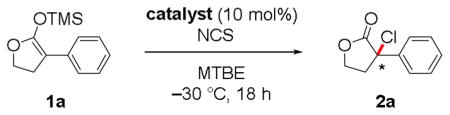				
entry	catalyst	Ar	yield (%)^b^	ee (%)^c^
1	**3**	9-phenanthryl	78	56
2	**4**	9-phenanthryl	>95	81
3	**5a**	9-phenanthryl	>95	86
4	**5b**	1-phenanthryl	>95	33
5	**5c**	3-phenanthryl	>95	84
6	**5d**	phenyl	>95	34
7	**5e**	2-naphthyl	>95	47
8	**5f**	4-pyrenyl	>95	62
9	**5g**	3-(*N*-methylcarbazolyl)	>95	47

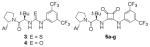				

a Conditions: **1a** (0.05 mmol), NCS (0.0375 mmol), catalyst (0.005 mmol) in MTBE (3 mL) under nitrogen at −30 °C for 18 hours.

b Yield based on NCS determined by ^1^H NMR analysis of crude reaction mixture.

c Enantiomeric excess determined by HPLC analysis on commercial chiral columns.

**Table 2:** Optimization of Reaction Parameters^a^

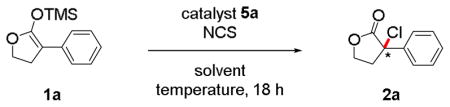				
entry	solvent	temperature (°C)	yield (%)^b^	ee (%)^c^
1	DCM	0	ND	0
2	toluene	0	ND	60
3	methyl cyclopentyl ether	0	ND	74
4	MTBE	0	>95	80
5	MTBE	−30	>95	86
6	MTBE	−78	>95	90
7	10% hexanes in MTBE	−30	>95	90
8	25% hexanes in MTBE	−30	64	30
9	hexanes	−30	18	0

a Conditions: **1a** (0.05 mmol), NCS (0.0375 mmol), **5a** (0.005 mmol) in solvent (3 mL) under nitrogen at indicated temperature for 18 hours.

b Yield based on NCS determined by ^1^H NMR analysis of crude reaction mixture.

c Enantiomeric excess determined by HPLC analysis on commercial chiral columns.

**Table 3:** Scope of Silyl Enolate Substrate^a^

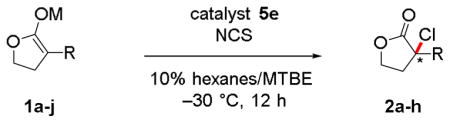					
substrate	product	M	R	yield (%)^b^	ee (%)^c^
**1a**	**2a**	TMS	C_6_H_5_	91	90
**1b**	**2b**	TMS	2-naphthyl	90	92
**1c**	**2c**	TMS	4-Br-C_6_H_4_	94	92
**1d**	**2d**	TMS	4-Cl-C_6_H_4_	96	94
**1e**	**2e**	TMS	4-CH_3_-C_6_H_4_	94	82
**1f**	**2f**	TMS	4-CH_3_O-C_6_H_4_	95	11
**1g**	**2g**	TMS	3-CH_3_O-C_6_H_4_	84	80
**1h**	**2h**	TMS	3-thienyl	88	58
**1i**	**2a**	TES	C_6_H_5_	90	86
**1j**	**2a**	TBS	C_6_H_5_	79	83

a Data represent the average of two experiments. Conditions: silyl enolate (0.2 mmol), NCS (0.4 mmol), **5e** (0.02 mmol) in MTBE (3 mL) and hexanes (0.3 mL) under nitrogen at −30 °C for 12 hours.

b Isolated yield of purified product.

c Enantiomeric excess determined by HPLC analysis on commercial chiral columns.
