# Supplementary material for: Enantioselective synthesis of tertiary α-chloro esters by non-covalent catalysis
Source: Tetrahedron Lett. Author manuscript; Available in PMC 2016 Jun 3. (PMC4465138; doi:10.1016/j.tetlet.2015.01.124)

Enantioselective Synthesis of Tertiary α-Chloro Esters by Non-Covalent Catalysis

Richard Y. Liu, Masayuki Wasa, Eric N. Jacobsen*

Department of Chemistry & Chemical Biology, Harvard University, Cambridge, MA 02138
United States

Supporting Information

Table of Contents

Procedures, Materials, and Instrumentation S-2

Experimental Section S-4

A. Catalyst Synthesis S-4

B. Substrate Synthesis S-8

C. Enantioselective Chlorination Reaction S-11

D. Substitution Reactions of α-Chloro Esters S-22

References S-28

^{1}H, ^{13}C, and ^{19}F NMR Spectra S-29

Procedures, Materials, and Instrumentation

General experimental procedures. All reactions were performed in standard, dry glassware fitted with rubber septa under an inert atmosphere of nitrogen unless otherwise described. Stainless steel syringes or cannulae were used to transfer air- and moisture-sensitive liquids. Reported concentrations refer to solution volumes at room temperature. Evaporation and concentration *in vacuo* was performed using house vacuum (ca. 40 mm Hg). Column chromatography was performed ZEOprep® 60 (40–63 micron) silica gel from American Scientific, eluting under positive pressure of nitrogen. Thin layer chromatography (TLC) was used for reaction monitoring and product detection using pre-coated glass plates covered with 0.20 mm silica gel with fluorescent indicator, visualized using UV light (λ_{ex} = 254 nm) or CAM stain.

Materials. Reagents were purchased in reagent grade from commercial suppliers and used without further purification, unless otherwise described. *N*-chlorosuccinimide was recrystallized from hot glacial acetic acid. Anhydrous solvents (toluene, *tert*-butyl methyl ether, Et_2O, CH_2Cl_2, CPME, hexanes) were prepared by passing the solvent through an activated alumina column. Triethylamine, diisoproylamine and diisopropylethylamine were distilled from CaH_2 at atmospheric pressure. H_2O, in synthetic procedures, refers to distilled water; brine refers to saturated aq. NaCl.

Instrumentation. Proton nuclear magnetic resonance (^1H NMR) spectra and proton-decoupled carbon nuclear magnetic resonance (^{13}C {^1H} NMR) spectra were recorded at 25 °C (unless stated otherwise) on Inova 600 (600 MHz) or Varian Unity/Inova 500 (500 MHz) spectrometers at the Harvard University nuclear magnetic resonance facility. Chemical shifts for protons are reported in parts per million downfield from tetramethylsilane and are referenced to residual protium in the NMR solvent. Chemical shifts for carbon are reported in parts per million downfield from tetramethylsilane and are referenced to the carbon resonances of the solvent. The solvent peak was referenced to 7.26 ppm for ^1H and 77.0 ppm for ^{13}C for $CDCl_3$, to 3.31 ppm for ^1H and 49.15 ppm for ^{13}C for CD_3OD, to 5.32 ppm for ^1H and 54.0 ppm for ^{13}C for CD_2Cl_2, and to 2.05 for ^1H and 39.5 ppm for ^{13}C for DMSO-d_6. Data are represented as follows: chemical shift, integration, multiplicity (br = broad, s = singlet, d = doublet, t = triplet, q = quartet, qn = quintet, sp = septet, m = multiplet), coupling

constants in Hertz (Hz). In the case of compounds containing one or more fluorine atom(s), it should be noted that ^{13}C NMR experiments were obtained without ^{19}F decoupling.

Optical rotations were measured using a 1 mL cell with a 5 cm path length on a Jasco P-2000 digital polarimeter.

Infrared spectra were recorded using a Bruker Tensor 27 FT-IR spectrometer. Data are represented as follows: frequency of absorption (cm^{-1}), intensity of absorption (s = strong, m = medium, w = weak, br = broad).

High-resolution mass spectrometry was measured using a Bruker microTOF-QII™ ESI-Qq-TOF mass spectrometer calibrated using an aqueous sodium formate solution (prepared via adding 1 mL of 1 M aq. NaOH in 100 mL of 1% aq. formic acid).

Chiral high performance liquid chromatography (HPLC) analysis was performed using an Agilent 1200 quaternary HPLC system with a commercially available AS-H, AD-H, IC, and OD-H chiral columns.

Abbreviations. Boc = *tert*-butoxycarbonyl, *n*-BuLi = *n*-butyllithium, CPME = cyclopentyl methyl ether, DCM = dichloromethane, DIPEA = diisopropylethylamine, ee = enantiomeric excess, ESI = electrospray ionization, Et_2O = diethyl ether, Et_3N = triethylamine, EtOAc = ethyl acetate, Fmoc = fluorenylmethoxycarbonyl, HR = high-resolution, HPLC = high performance liquid chromatography, LDA = lithium diisopropylamide, LiHMDS = ;ithium bis(trimethylsilyl)amide, MS = mass spectrometry, NA = not applicable, NCS = *N*-chlorosuccinimide, RT = room temperature, TBME = *tert*-butylmethyl ether, THF = tetrahydrofuran, TOF = time-of-flight.

Experimental Section

A. Catalyst Synthesis

General Procedure for the Preparation of Chiral Squaramide Catalysts

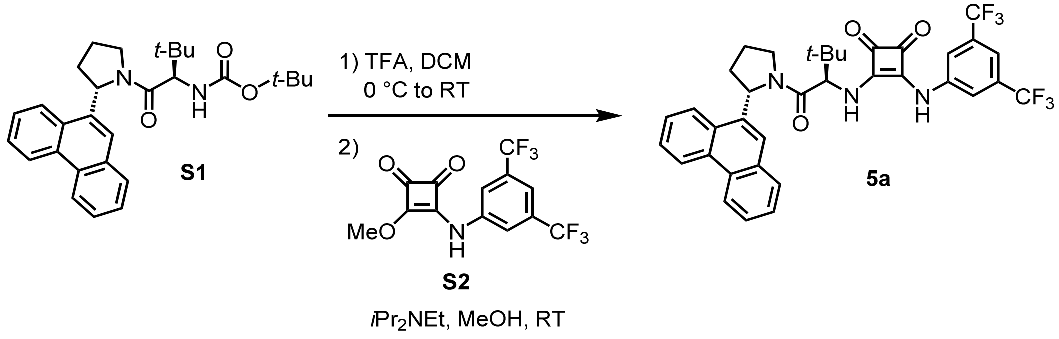

3-((3,5-Bis(trifluoromethyl)phenyl)amino)-4-(((*R*)-3,3-dimethyl-1-oxo-1-((*S*)-2-(phenanthren-9-yl)pyrrolidin-1-yl)butan-2-yl)amino)cyclobut-3-ene-1,2-dione (5a)

In a 25 mL round bottom flask, cooled to 0 °C and equipped with a magnetic stir bar, **S1**[1] (178 mg, 0.36 mmol) in dichloromethane was slowly added to a 30% v/v mixture of TFA in DCM (4 mL total). The flask was allowed to warm to room temperature over the course of 1 hr, and the reaction mixture was then concentrated to yield a translucent, yellow foam. Methanol (4 mL) and diisopropylethylamine (0.125 mL, 0.72 mmol) were added at room temperature and the mixture stirred vigorously. After 10 minutes, **S2**[2] (122 mg, 0.36 mmol) was added as a solid in a single portion, and the reaction was stirred under nitrogen for 12 hr. The solvent was removed *in vacuo* and flash chromatographed on silica, eluting with 30% EtOAc in hexanes to give **5a** as a slightly yellow foam. Recrystallization from MeOH/hexanes yields an off-white powder (151 mg, 63% overall yield). ^{1}H NMR (600MHz, DMSO-d_6, an 8:1 mixture of rotamers; major rotamer resonances listed): δ = 10.39 (s, 1 H), 8.88 – 8.81 (m, 1 H), 8.74 (d, J = 8.5 Hz, 1 H), 8.26 – 8.16 (m, 2 H), 8.08 – 8.03 (m, 2 H), 7.73 – 7.63 (m, 4 H), 7.59 – 7.50 (m, 1 H), 7.29 (s, 1 H), 7.28 – 7.22 (m, 1 H), 5.89 (d, J = 8.2 Hz, 1 H), 5.14 (d, J = 10.0 Hz, 1 H), 4.33 – 4.23 (m, 1 H), 3.93 – 3.83 (m, 1 H), 3.38 – 3.31 (m, 1 H), 2.55 – 2.45 (m, 2 H), 2.08 – 1.99 (m, 1 H), 1.97 – 1.88 (m, 1 H), 1.87 – 1.81 (m, 1 H), 1.09 (s, 9 H). ^{13}C NMR (126 MHz, DMSO-d_6): δ = 185.4, 181.5, 170.0, 168.7, 163.6, 141.7, 136.5, 132.3, 132.0, 131.6, 130.9, 129.9, 129.9, 128.9, 127.6, 127.2, 127.0, 124.9, 124.9, 124.2, 123.3, 122.8, 122.3, 118.7, 62.3, 58.3, 48.6, 36.0, 32.6, 26.5, 24.0. FTIR (neat): 3262, 2963, 1605, 1557, 1477, 1429, 1379, 1278, 1182, 1135, 750 cm^{-1}. HRMS (ESI): $[M+H]^+$ calculated for $C_{36}H_{32}F_6N_3O_3$: 668.2342. Found: 668.2350.

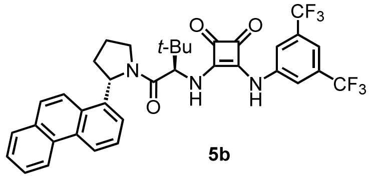

5b

3-((3,5-Bis(trifluoromethyl)phenyl)amino)-4-(((*R*)-3,3-dimethyl-1-oxo-1-((*S*)-2-(phenanthren-1-yl)pyrrolidin-1-yl)butan-2-yl)amino)cyclobut-3-ene-1,2-dione (5b)

White powder, synthesized according to the general procedure. ^{1}H NMR (500MHz, DMSO-d_6, a 7:1 mixture of rotamers; major rotamer resonances listed): δ = 10.97 (s, 1 H), 9.36 (d, J = 8.7 Hz, 1 H), 9.24 (d, J = 8.5 Hz, 1 H), 8.81 (m, 1 H), 8.66 (d, J = 9.0 Hz, 1 H), 8.67 – 8.59 (br s, 2 H), 8.54 (d, J = 8.2 Hz, 1 H), 8.46 (d, J = 8.9 Hz, 1 H), 8.28 – 8.19 (br s, 2 H), 8.17 (m, 1H), 8.07 (m, 1H), 7.75 (d, J = 6.7 Hz, 1 H), 6.49 (d, J = 8.3 Hz, 1 H), 5.67 (m, 1 H), 4.70 (m, 1 H), 2.56 (m, 1 H), 2.44 (m, 1 H), 2.34 (m, 1 H), 1.77 (m, 2 H), 1.19 (s, 9 H). HRMS (ESI): $[M+H]^+$ calculated for $C_{36}H_{32}F_6N_3O_3$: 668.2342. Found: 668.2330.

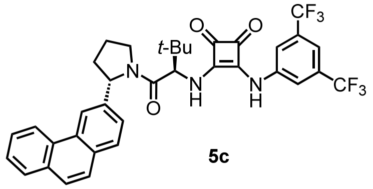

5c

3-((3,5-Bis(trifluoromethyl)phenyl)amino)-4-(((*R*)-3,3-dimethyl-1-oxo-1-((*S*)-2-(phenanthren-3-yl)pyrrolidin-1-yl)butan-2-yl)amino)cyclobut-3-ene-1,2-dione (5c)

White powder, synthesized according to the general procedure. ^{1}H NMR (500MHz, DMSO-d_6, a 2.4:1 mixture of rotamers; major rotamer resonances listed): δ = 10.24 (s, 1 H), 8.90 (br s, 1 H), 8.52 (m, 1 H), 8.31 (d, J = 11.0 Hz, 1H), 8.23 (d, J = 10.0 Hz, 1 H), 8.08 (m, 1 H), 8.02 – 7.98 (m, 1 H), 7.91 (s, 2 H), 7.87 (d, J = 8.2 Hz, 1 H), 7.76 – 7.71 (m, 2 H), 7.65 (m, 1 H), 7.47 (m, 1 H), 7.35 (m, 1 H), 7.29 (m, 1 H), 5.34 (dd, J = 8.1, 3.0 Hz, 1 H), 5.10 (d, J = 10.0 Hz, 1 H), 4.24 (m, J = 10 Hz, 1 H), 3.86 – 3.81 (m, 1 H), 3.30 (m, 1 H), 2.39 (m, 1 H), 2.01 – 1.86 (m, 3 H), 1.03 (s, 9H). FTIR (neat): 2937, 1614, 1461, 1389, 1350, 1202, 1154, 1023, 909, 722 cm^{-1}. HRMS (ESI): $[M+H]^+$ calculated for $C_{36}H_{32}F_6N_3O_3$: 668.2342. Found: 668.2349.

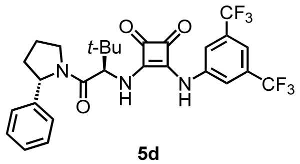

5d

3-((3,5-Bis(trifluoromethyl)phenyl)amino)-4-(((*R*)-3,3-dimethyl-1-oxo-1-((*S*)-2-phenylpyrrolidin-1-yl)butan-2-yl)amino)cyclobut-3-ene-1,2-dione (5d)

White powder, synthesized according to the general procedure. ^{1}H NMR (500MHz, DMSO-d_6, a 2.3:1 mixture of rotamers; major rotamer resonances listed): δ = 10.45 – 10.39 (s, 1 H), 8.32 – 8.22 (m, 1 H), 8.10 – 8.03 (m, 2 H), 7.62 (br. s., 1 H), 7.45 – 7.35 (m, 1 H), 7.26 – 7.18 (m, 2 H), 7.17 – 7.07 (m, 2 H), 5.17 – 5.07 (m, 1 H), 5.03 (d, *J* = 10.0 Hz, 1 H), 4.05 – 3.95 (m, 1 H), 3.80 – 3.70 (m, 1 H), 3.70 – 3.61 (m, 1 H), 2.30 – 2.20 (m, 1 H), 1.95 – 1.85 (m, 1 H), 1.79 – 1.69 (m, 1 H), 1.02 (s, 9 H). ^{13}C NMR (126MHz, DMSO-d_6): δ = 185.1, 181.1, 169.7, 168.5, 163.2, 143.9, 141.8, 132.2, 131.9, 129.1, 128.8, 127.4, 126.9, 126.0, 124.9, 122.7, 118.7, 61.9, 61.0, 48.6, 36.4, 34.5, 26.4, 26.3, 23.7. FTIR (neat): 3275, 2964, 1609, 1558, 1483, 1435, 1380, 1278, 1182, 1136, 701 cm^{-1}. HRMS (ESI): [M+H]$^+$ calculated for $C_{28}H_{28}F_6N_3O_3$: 568.2029. Found: 568.2039.

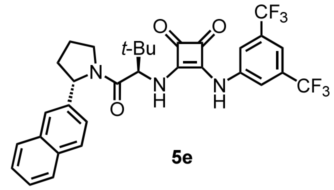

5e

3-((3,5-Bis(trifluoromethyl)phenyl)amino)-4-(((*R*)-3,3-dimethyl-1-oxo-1-((*S*)-2-(naphthen-2-yl)pyrrolidin-1-yl)butan-2-yl)amino)cyclobut-3-ene-1,2-dione (5e)

White powder, synthesized according to the general procedure. ^{1}H NMR (500MHz, DMSO-d_6, a 2:1 mixture of rotamers; major rotamer resonances listed); δ = 10.41 (s, 1 H), 8.25 (d, *J* = 10.0 Hz, 1 H), 8.06 (s, 2 H), 7.88 (d, *J* = 7.7 Hz, 1 H), 7.80 (d, *J* = 7.8 Hz, 1 H), 7.71 (s, 1 H), 7.51 – 7.46 (m, 2 H), 7.41 (d, *J* = 10.2 Hz, 1H), 7.02 (t, *J* = 7.0 Hz, 1 H), 5.22 (dd, *J* = 8.0, 4.0 Hz), 5.04 (d, *J* = 10.0 Hz), 4.47 (d, *J* = 9.6 Hz), 4.01 – 3.94 (m, 1 H), 3.90 – 3.80 (m, 1 H), 3.56 – 3.51 (m, 1 H), 2.48 – 2.42 (m, 1 H), 2.37 – 2.27 (m, 1 H), 2.06 – 1.93 (m, 2 H), 1.92 – 1.79 (m, 2 H), 1.00 (s, 9 H). FTIR (neat): 2954, 1770, 1600, 1512, 1444, 1377, 1251, 1168, 1020, 840, 698, 523 cm^{-1}. HRMS (ESI): [M+H]$^+$ calculated for $C_{32}H_{30}F_6N_3O_3$: 618.2191. Found: 618.3002.

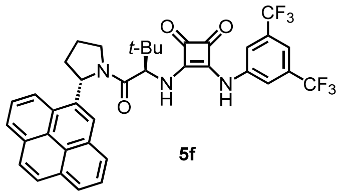

5f

3-((3,5-Bis(trifluoromethyl)phenyl)amino)-4-(((*R*)-3,3-dimethyl-1-oxo-1-((*S*)-2-(pyren-4-yl)pyrrolidin-1-yl)butan-2-yl)amino)cyclobut-3-ene-1,2-dione (5f)

Yellow powder, synthesized according to the general procedure. ^{1}H NMR (500MHz, DMSO-*d$_6$*, a 7:1 mixture of rotamers; major rotamer resonances listed): δ = 10.40 – 10.37 (s, 1 H), 8.47 (d, *J* = 8.0 Hz, 1 H), 8.30 (m, 2 H), 8.16 (m, 3 H), 8.09 – 8.05 (m, 3 H), 8.03 – 7.95 (m, 1 H), 7.75 – 7.65 (m, 2 H), 6.09 – 6.04 (m, 1 H), 5.20 (d, *J* = 10.0 Hz, 1 H), 4.40 – 4.33 (m, 1 H), 3.97 – 3.89 (m, 1 H), 2.63 – 2.53 (m, 1 H), 2.15 – 1.86 (m, 3 H), 1.10 (s, 9 H). ^{13}C NMR (126MHz, DMSO-*d$_6$*): δ = 185.5, 181.5, 170.1, 168.8, 163.7, 141.7, 137.5, 132.3, 132.0, 131.8, 131.0, 130.7, 129.2, 128.2, 127.8, 126.7, 126.5, 125.9, 125.7, 125.4, 125.0, 124.9, 123.6, 122.8, 122.6, 122.1, 118.7, 62.3, 58.5, 48.7, 36.1, 32.7, 26.6, 24.1. FTIR (neat): 3275, 2972, 1606, 1558, 1476, 1429, 1379, 1278, 1182, 1136, 760 cm^{-1}. HRMS (ESI): [M+H]$^{+}$ calculated for $C_{38}H_{32}F_6N_3O_3$: 692.2342. Found: 692.2334.

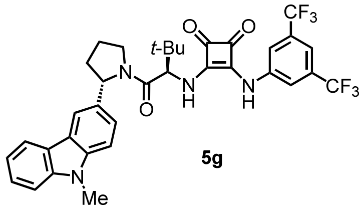

5g

3-((3,5-Bis(trifluoromethyl)phenyl)amino)-4-(((*R*)-3,3-dimethyl-1-oxo-1-((*S*)-2-(9-methyl-9*H*-carbazol-3-yl)pyrrolidin-1-yl)butan-2-yl)amino)cyclobut-3-ene-1,2-dione (5g)

Light yellow foam, synthesized according to the general procedure. ^{1}H NMR (500MHz, DMSO-*d$_6$*, a 3:1 mixture of rotamers; major rotamer resonances listed): δ = 10.38 (s, 1 H), 8.33 – 8.26 (m, 2 H), 8.15 – 8.02 (m, 4 H), 7.64 (br s, 1 H), 7.44 (m, 1 H), 7.25 (m, 1 H), 7.22 (m, 1 H), 6.99 (m, 1 H), 5.30 (m, 2 H), 5.08 (d, *J* = 9.9 Hz, 1 H), 4.66 (d, *J* = 10.0 Hz, 1 H), 3.72 (s, 3 H), 2.37 – 2.33 (m, 1 H), 1.99 – 1.83 (m, 2 H), 1.27 – 1.21 (m, 2 H), 1.05 (s, 9 H). ^{13}C NMR (126 MHz, DMSO-*d$_6$*): δ = 184.5, 181.1, 170.9, 169.4, 163.2, 141.5, 141.1, 131.9, 131.6, 125.7, 124.6, 122.5, 122.2, 120.7, 118.6, 115.3, 109.6, 105.9, 64.7, 61.9, 61.3, 52.6, 48.4, 35.9, 35.7, 26.2, 26.1, 25.9. FTIR (neat): 2937, 1604, 1461, 1272, 1194, 1153,

1034, 909, 879, 827, 731. cm^{-1}. HRMS (ESI): $[M+H]^+$ calculated for $C_{36}H_{32}F_6N_3O_3$: 671.2457. Found: 671.2450.

B. Substrate Synthesis

General Procedure for the Preparation of Silyl Ketene Acetal Substrates

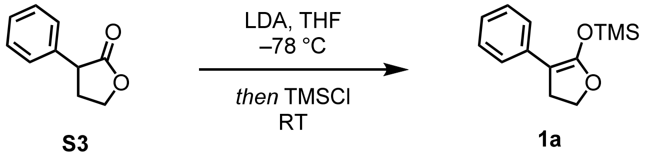

((3-Phenyl-4,5-dihydrofuran-2-yl)oxy)trimethylsilane (1a)

According to a previously reported procedure,[4] a solution of *n*-butyllithium (2.5 M in hexanes, 8.4 mL, 21.0 mmol) was added to a THF (25 mL) solution of diisopropylamine (2.85 mL, 21.0 mmol) at –78 °C in a 100 mL round-bottom flask. The solution was stirred for 5 minutes, and a 2 M solution of **S3** (3.25 g, 20 mmol) was added dropwise by cannula. After stirring at –78 °C for another 30 minutes, chlorotrimethylsilane (2.7 mL, 21 mmol) was added by syringe, and the flask was warmed to room temperature over the course of 3 hr. Following removal of solvent *in vacuo*, the crude mixture was suspended in 30 mL of hexanes and quickly passed through a short plug of Celite. The crude filtrate was concentrated and distilled under reduced pressure to yield a clear, slightly yellow oil (2.6 g, 56% yield), which solidified upon standing. The spectral properties matched those in the prior report.[4] The silyl ketene acetals were stored in the glovebox under inert atmosphere.

Substrates **1d**, **1e**, **1f,** and **1h** were also synthesized according to the general procedure and were spectroscopically identical to the compounds in our previous report.[4]

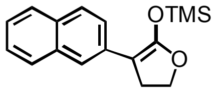

1b

((3-(Naphthalen-2-yl)-4,5-dihydrofuran-2-yl)oxy)trimethylsilane (1b)

Light yellow solid, synthesized according to the general procedure. ^{1}H NMR (600MHz, $CDCl_3$): δ = 7.92 (ddd, J = 12.8, 8.7, 1.8 Hz, 1 H), 7.85 (m, 1 H), 7.81 – 7.76 (m, 3 H), 7.54 – 7.52 (m, 1 H), 7.45 (m, 1 H), 7.37 (m, 1 H), 4.45 (d, J = 8.8 Hz, 2 H), 3.12 (d, J = 8.8 Hz, 2 H), 0.25 (s, 9 H). ^{13}C NMR (126 MHz, $CDCl_3$): δ = 155.7, 134.1, 130.6, 130.5, 127.5, 127.4, 127.3, 127.2, 125.8, 124.0, 123.9, 120.4, 82.7, 66.5, 30.4, 0.9. FTIR (neat): 1667, 1599, 1440, 1380, 1120, 971, 842, 811, 767, 691 cm^{-1}.

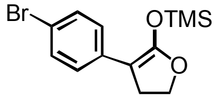

1c

((3-(4-Bromophenyl)-4,5-dihydrofuran-2-yl)oxy)trimethylsilane (1c)

White solid, synthesized according to the general procedure. ^{1}H NMR (600MHz, $CDCl_3$): δ = 7.39 (d, J =8.4 Hz, 2 H), 7.29 (d, J = 7.6 Hz, 2 H), 4.39 (d, J = 8.8 Hz, 2 H), 2.99 (d, J = 8.9 Hz, 2 H), 0.35 (s, 9 H). ^{13}C NMR (126 MHz, $CDCl_3$): δ = 155.2, 135.9, 131.0, 128.2, 123.6, 122.9, 82.1, 66.5, 30.1, 0.5. FTIR (neat): 1680, 1512, 1370, 1233, 1082, 990, 844, 809, 720, 619 cm^{-1}.

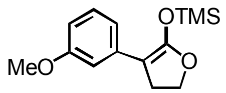

1g

((3-(3-(Methoxy)phenyl)-4,5-dihydrofuran-2-yl)oxy)trimethylsilane (1g)

Off-white solid, synthesized according to the general procedure. ^{1}H NMR (600MHz, $CDCl_3$): δ = 7.19 (td, J = 8.0, 2.5 Hz, 1 H), 7.04 (m, 1 H), 6.93 (m, 1 H), 6.60 (m, 1 H), 4.37 (t, J = 8.9 Hz, 2 H), 3.82 (s, 3 H), 2.98 (t, J = 8.8 Hz, 2 H), 0.36 (s, 9 H). ^{13}C NMR (126 MHz, $CDCl_3$): δ = 159.6, 137.3, 129.0, 116.3, 108.9, 82.3, 66.5, 54.9, 30.1, 0.4. FTIR (neat): 1670, 1370, 1512, 1244, 1190, 1147, 998, 846, 812, 761, 650 cm^{-1}.

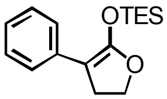

1i

Triethyl(3-phenyl-4,5-dihydrofuran-2-yl)oxy)silane (1i)

White solid, synthesized according to the general procedure, using TESCl instead of TMSCl. ^{1}H NMR (600MHz, $CDCl_3$): ^{1}H NMR (600MHz, $CDCl_3$): 7.50 (m, J = 8.4, 2 H), 7.28 (m, 2 H), 7.05 (m, 1 H), 4.29 (t, J = 8.9 Hz, 2 H), 2.90 (t, J = 9.1 Hz, 2 H), 1.06 (q, J = 7.5 Hz, 6 H), 0.95 – 0.81 (t, J = 7.3 Hz, 9 H). ^{13}C NMR (126 MHz, $CDCl_3$): δ = 155.5, 135.6, 128.8, 123.7, 129.9, 84.0, 66.4, 30.5, 14.9, 6.0. FTIR (neat): 1641, 1488, 1125, 1022, 953, 840, 761, 680 cm^{-1}.

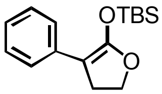

1j

***tert*-Butyldimethyl(3-phenyl-4,5-dihydrofuran-2-yl)oxy)silane (1j)**

White solid, synthesized according to the general procedure, using TBSCl instead of TMSCl. ^{1}H NMR (600MHz, $CDCl_3$): 7.41 (dt, J = 8.4, 1.5 Hz, 2 H), 7.29 (m, 2 H), 7.03 (m, 1 H), 4.38 (t, J = 8.9 Hz, 2 H), 2.99 (t, J = 8.9 Hz, 2 H), 1.01 (s, 9 H), 0.29 (s, 6 H). ^{13}C NMR (126 MHz, $CDCl_3$): δ = 155.4, 135.9, 128.2, 123.8, 123.0, 82.1, 66.5, 30.3, 25.8, –3.9. FTIR (neat): 1601, 1512, 1250, 1155, 1020, 953, 839, 754, 698 cm^{-1}.

C. Enantioselective Chlorination Reaction

Experimental Procedure for Reaction Optimization

Under dry nitrogen atmosphere, flame-dried 2 dram vial equipped with a screw-top septum cap and a magnetic stir bar was charged with NCS (0.75 – 3 equiv.), catalyst (0.1 equiv.), and any additives. Solvent (0.5 – 6 mL) was added, and the mixture was cooled to –78 °C for 20 minutes. A 0.05 M solution of silyl ketene acetal **1a** (1 mL, 0.05 mmol, 1 equiv.), pre-cooled to –78 °C, was added by syringe, and the vial was transferred to a cooling bath of appropriate temperature. Upon completion, the solvent was removed and yield was determined by ^{1}H NMR analysis of the crude sample using 0.5 equiv of dibromomethane as internal standard. The crude mixture was concentrated again to ~0.5 mL and loaded directly onto silica for preparative TLC. The plate was eluted with 40% EtOAc in hexanes to separate pure **2a**, which was analyzed by chiral HPLC for determination of enantiomeric excess.

Table SI-1. Additional catalyst screening data

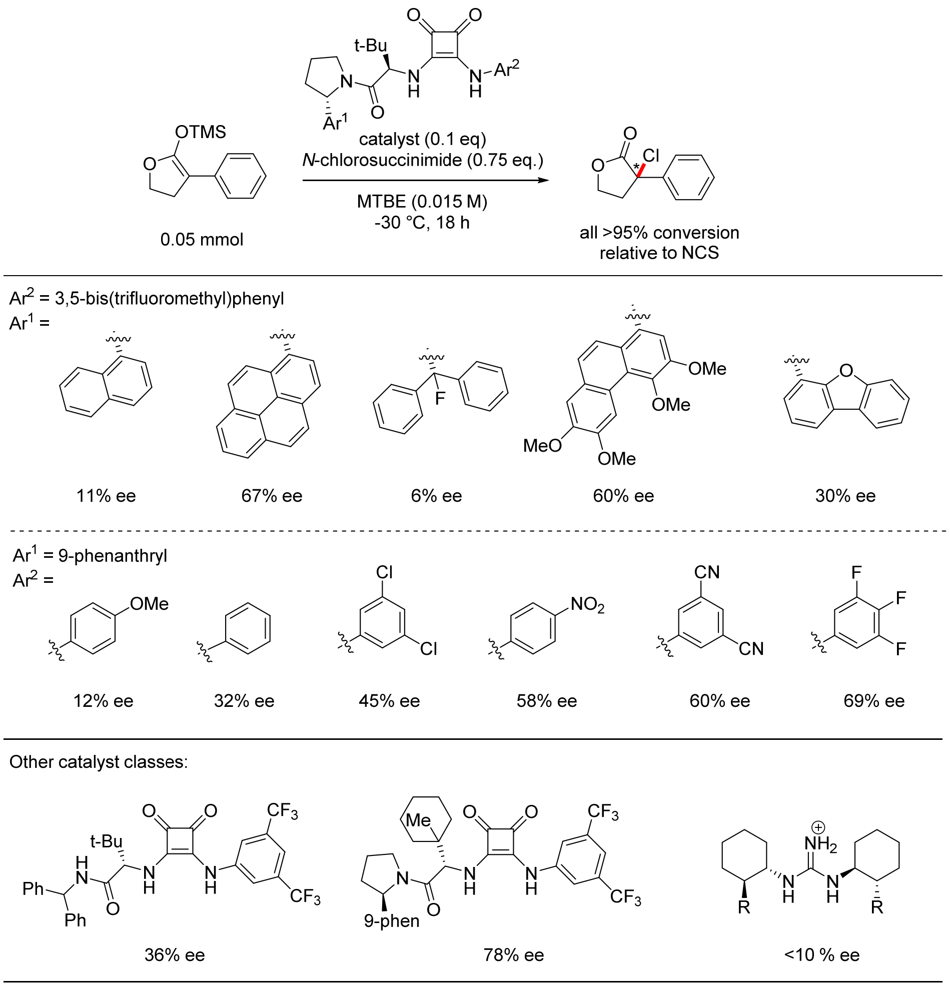

Table SI-2. Selected results with suboptimal substrates

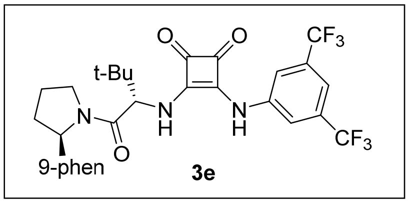

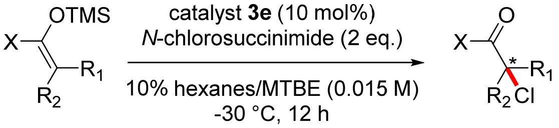

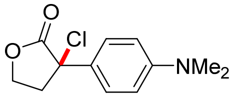

[a]31% yield
22% ee

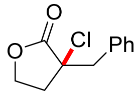

>95% yield
10% ee

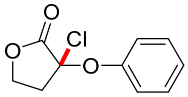

>95% yield
0% ee

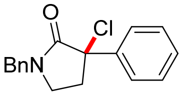

>95% yield
0% ee

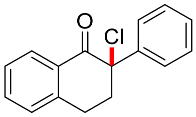

>95% yield
5% ee

[a] Reaction performed using single equivalent of *N*-chlorosuccinimide to reduce competing arene chlorination.

Table SI-3. Use of alternative electrophilic halogenation reagents

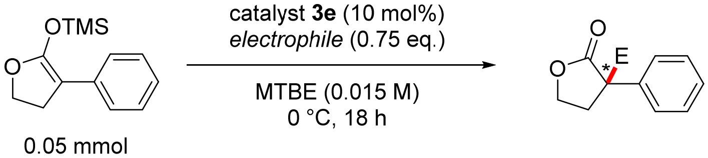

0.05 mmol

electrophile	yield	ee
N-chlorosuccinimide	>95%	86%
N-bromosuccinimide	90%	15%
N-iodosuccinimide	NR	ND
N-chlorophthalimide	>95%	53%
N-fluorobenzenesulfonimide	39%	8%

General Procedure for Enantioselective Chlorination Reaction

Under dry nitrogen atmosphere, an oven-dried 25 mL round-bottom flask equipped with a rubber septum and a magnetic stir bar was charged with NCS (53.4 mg, 0.4 mmol, 2 equiv.) and catalyst **3e** (13.3 mg, 0.02 mmol, 0.1 equiv.). Hexanes (1.2 mL) and MTBE (10 mL) were added, and the mixture was cooled to –78 °C for 20 minutes. A 0.1 M solution of silyl ketene acetal (2 mL, 0.2 mmol, 1 equiv.), pre-cooled to –78 °C, was added dropwise, and the flask was transferred to a cooling bath at –30 °C. After stirring for 12 h, the reaction mixture was concentrated to ca. 3 mL and loaded directly onto a silica column. Elution with a gradient of 10% EtOAc in hexanes to pure EtOAc yielded the desired product after solvent removal. The enantiomeric excess was determined by chiral HPLC analysis.

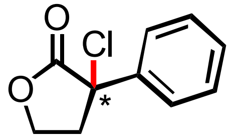

2a

3-Chloro-3-phenyldihydrofuran-2(3*H*)-one (2a)

Substrate **1a** was chlorinated following the general procedure. **2a** was obtained as a colorless oil (36 mg, 91% yield, 90% ee). $[\alpha]^{25}_{D}$ = –2.2° (c = 1.0, $CHCl_3$). ^{1}H NMR (600 MHz, $CDCl_3$): δ = 7.69 – 7.66 (m, 2 H), 7.44 – 7.37 (m, 3 H), 4.58 (td, *J* = 8.9, 5.9 Hz, 1 H), 4.43 (ddd, *J* = 9.1, 7.3, 2.9 Hz, 1 H), 3.01 – 2.91 (m, 2 H). ^{13}C NMR (126 MHz, $CDCl_3$): δ = 175.2, 139.3, 131.9, 131.4, 129.6, 68.3, 68.0, 43.2. IR (neat): 2930, 1790, 1448, 1372, 1214, 1162, 1073, 746, 694, 641, 601, 567 cm^{-1}. HRMS (ESI): $[M+Na]^+$ calculated for $C_{10}H_9ClO_2$: 219.0189. Found: 219.0180.

HPLC (ChiralPak IC, 10% *i*PrOH in hexanes, 1 mL/min, 254 nm)

racemic

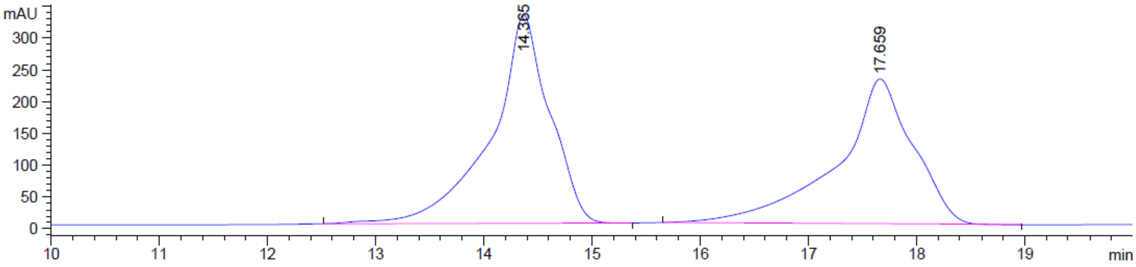

enantioenriched

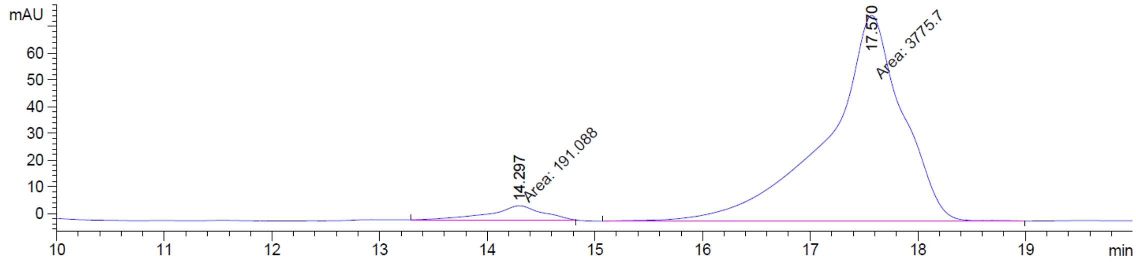

Peak	Retention time (min)	Area (mAU*s)	Area %
1	14.32	191.09	4.82
2	17.62	3775.7	95.18

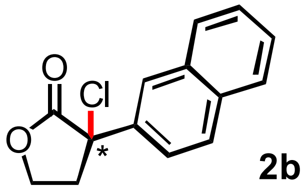

2b

3-Chloro-3-(naphthalen-2-yl)dihydrofuran-2(3*H*)-one (2b)

Substrate **1b** was chlorinated following the general procedure. **2b** was obtained as an off-white powder (44 mg, 90% yield, 92% ee). $[\alpha]^{25}_{D}$ = –1.4° (c = 1.0, $CHCl_3$). ^{1}H NMR (600 MHz, $CDCl_3$): δ = 8.12 (d, *J* = 1.9 Hz, 1 H), 7.92 (dd, *J* = 8.6, 2.4 Hz, 1 H), 7.86 (m, 2 H), 7.77 (m, 1 H), 7.54 (m, 2 H), 4.66 – 4.61 (m, 1 H), 4.47 (m, *J* = 9.0 Hz, 1 H), 3.13 – 3.01 (m, 2 H). ^{13}C NMR (125 MHz, $CDCl_3$): δ = 172.4, 133.7, 133.2, 132.6, 128.9, 128.5, 127.6, 127.3, 126.8, 126.7, 127.1, 124.4, 65.8, 65.3, 40.7. IR (neat): 2940, 1789, 1374, 1160, 1073, 1050, 1021, 991, 869, 746, 694, 602, 568 cm^{-1}. HRMS (ESI): $[M+Na]^+$ calculated for $C_{14}H_{11}ClO_2$: 269.0345. Found: 269.0343.

HPLC (ChiralPak IC, 10% *i*PrOH in hexanes, 1 mL/min, 254 nm)

racemic

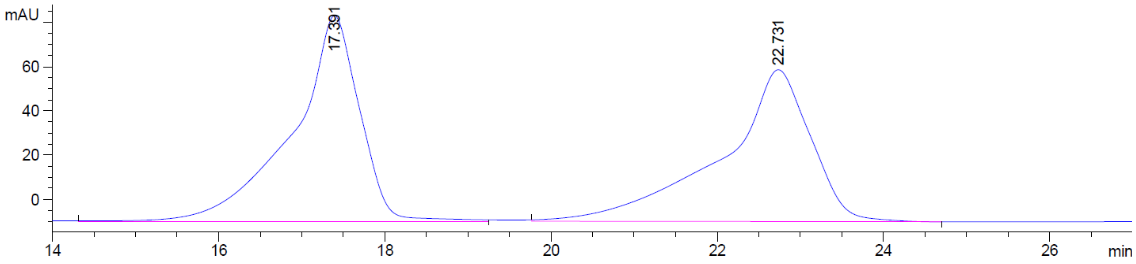

enantioenriched

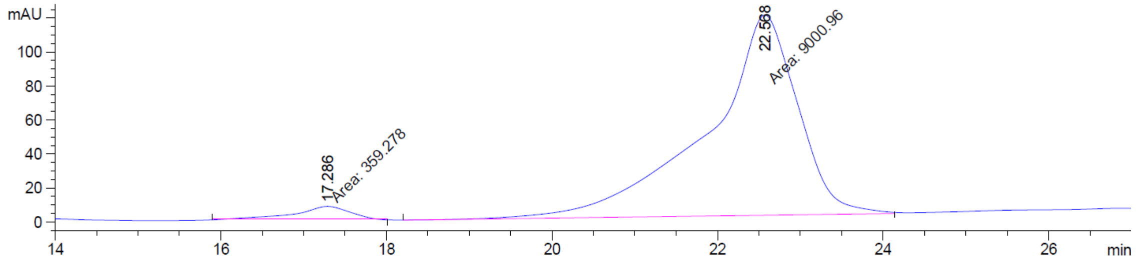

Peak	Retention time (min)	Area (mAU*s)	Area %
1	17.34	359.28	3.84
2	22.66	9001.0	96.16

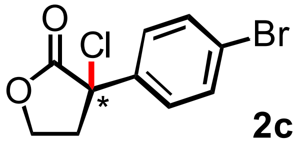

2c

3-Chloro-3-(4-bromophenyl)dihydrofuran-2(3*H*)-one (2c)

Substrate **1c** was chlorinated following the general procedure. **2c** was obtained as a colorless oil (52 mg, 94% yield, 92% ee). $[\alpha]^{25}_{D} = -3.9°$ (c = 1.0, $CHCl_3$). ^1H NMR (500 MHz, $CDCl_3$): δ = 7.68 – 7.67 (m, 2 H), 7.42 – 7.38 (m, 2 H), 4.57 (m, 1 H), 4.43 (m, 1 H), 2.98 – 2.93 (m, 2 H). ^{13}C NMR (125 MHz, $CDCl_3$): δ = 175.2, 139.2, 134.5, 131.4, 129.6, 68.3, 68.0, 43.3. IR (neat): 2918, 1790, 1371, 1214, 1162, 1094, 1074, 1021, 950, 694, 641, 600, 568 cm^{-1}. HRMS (ESI): $[M+Na]^+$ calculated for $C_{10}H_8BrClO_2$: 274.9474. Found: 274.9469.

HPLC (ChiralPak OD-H, 5% *i*PrOH in hexanes, 1 mL/min, 254 nm)

racemic

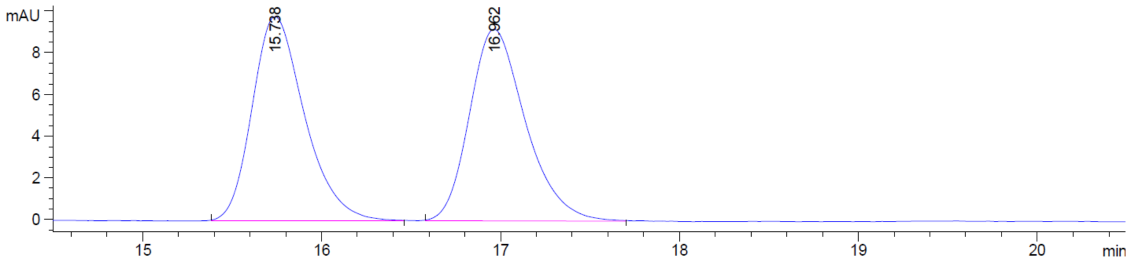

enantioenriched

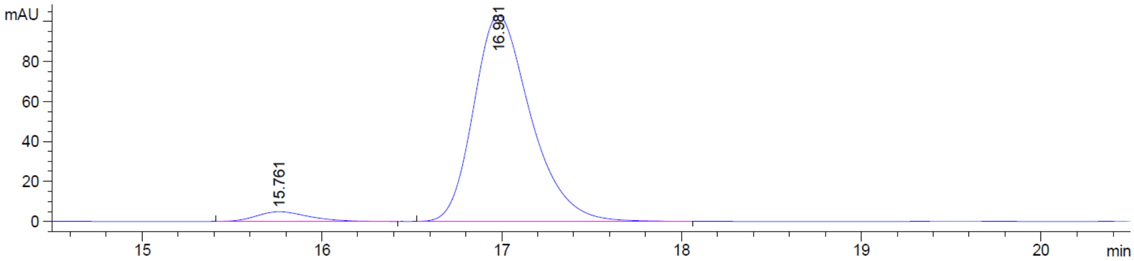

Peak	Retention time (min)	Area (mAU*s)	Area %
1	15.74	100.06	4.21
2	16.97	2275.0	95.79

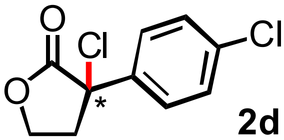

2d

3-Chloro-3-(4-chlorophenyl)dihydrofuran-2(3*H*)-one (2d)

Substrate **2d** was chlorinated following the general procedure. **3d** was obtained as a colorless oil (44 mg, 96% yield, 94% ee). $[\alpha]^{25}_{D}$ = –3.5° (c = 1.0, $CHCl_3$). ^{1}H NMR (600 MHz, $CDCl_3$): δ = 7.61 (d, *J* = 8.7 Hz, 2 H), 7.38 (d, *J* = 8.8 Hz, 2 H), 4.56 (dt, *J* = 8.8, 4.3 Hz), 4.44 (dt, *J* = 6.0, 3.0 Hz, 1 H), 2.93 – 2.91 (m, 2 H). ^{13}C NMR (125 MHz, $CDCl_3$): δ = 174.8, 138.0, 137.8, 131.6, 131.2, 68.0, 67.4, 43.0. IR (neat): 2980, 1773, 1445, 1333, 1214, 1168, 1070, 694, 599 cm^{-1}. HRMS (ESI): $[M+H]^+$ calculated for $C_{10}H_8Cl_2O_2$: 230.9980. Found: 230.9972.

HPLC (ChiralPak IC, 10% *i*PrOH in hexanes, 1 mL/min, 254 nm)

racemic

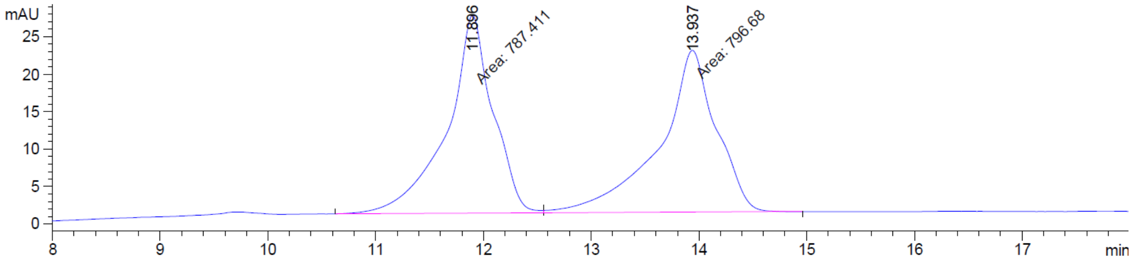

enantioenriched

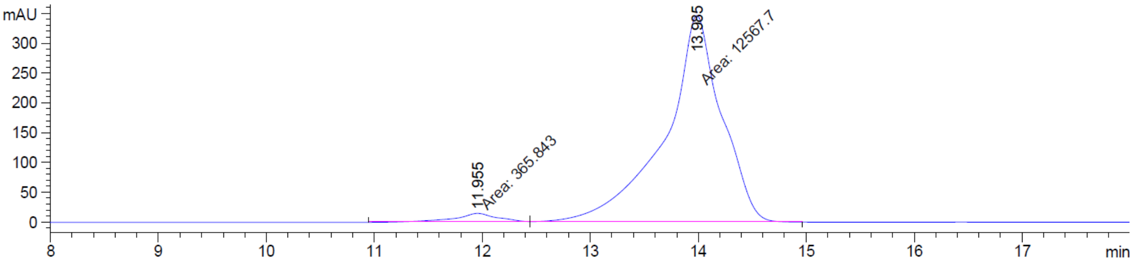

Peak	Retention time (min)	Area (mAU*s)	Area %
1	11.93	365.84	2.83
2	13.96	12567	97.17

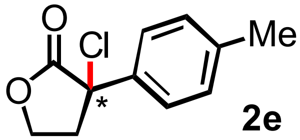

2e

3-Chloro-3-(4-methylphenyl)dihydrofuran-2(3*H*)-one (2e)

Substrate **1e** was chlorinated following the general procedure. **2e** was obtained as a colorless oil (40 mg, 94% yield, 82% ee). $[\alpha]^{25}_{D}$ = –1.7° (c = 1.0, $CHCl_3$). ^{1}H NMR (600 MHz, $CDCl_3$): δ = 7.57 (m, 2 H), 7.25 (m, 2 H), 4.57 (td, J = 8.9 Hz, 5.7 Hz), 4.41 (m, J = 9.0 Hz, 1 H), 3.00 – 2.90 (m, 2 H), 2.35 (s, 3 H). ^{13}C NMR (126 MHz, $CDCl_3$): δ = 172.6, 139.4, 133.6, 129.4, 126.9, 66.1, 65.3, 40.6, 21.1. IR (neat): 2980, 1768, 1442, 1370, 1151, 1068, 990, 778, 696 cm^{-1}. HRMS (ESI): $[M+H]^+$ calculated for $C_{11}H_{11}ClO_2$: 211.0525. Found: 211.0527.

HPLC (ChiralPak IC, 10% *i*PrOH in hexanes, 1 mL/min, 254 nm)

racemic

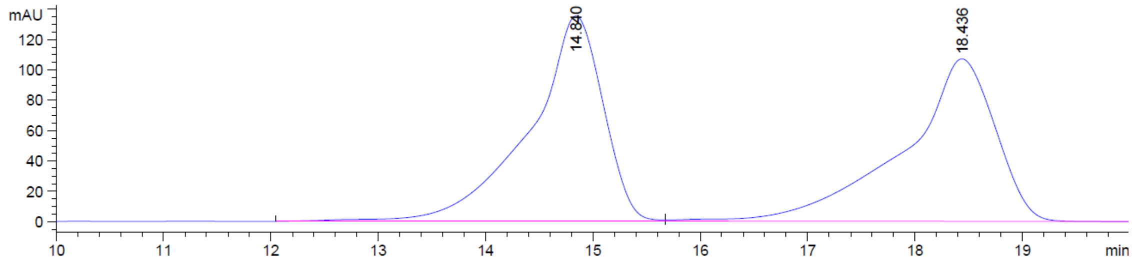

enantioenriched

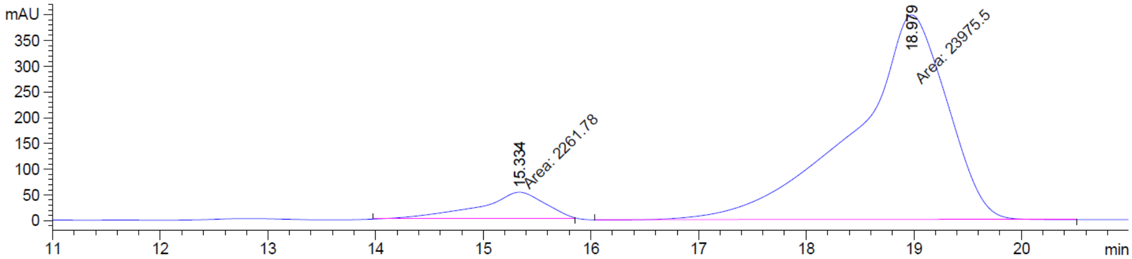

Peak	Retention time (min)	Area (mAU*s)	Area %
1	15.07	2261.8	8.62
2	19.21	23976	91.38

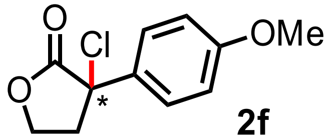

2f

3-Chloro-3-(4-methoxyphenyl)dihydrofuran-2(3*H*)-one (2f)

Substrate 1**f** was chlorinated following the general procedure. **2f** was obtained as a colorless oil (21 mg, 48% yield, 11% ee). $[\alpha]^{25}_{D}$ = –0.4° (c = 1.0, $CHCl_3$). ^1H NMR (600 MHz, $CDCl_3$): δ = 7.40 – 7.38 (m, 2 H), 6.94 – 6.91 (m, 2 H), 4.45 (ddd, *J* = 9.1, 8.1, 3.0 Hz, 1 H), 4.15 (td, *J* = 9.2, 6.1 Hz, 1 H), 3.81 (s, 3 H), 2.71 (ddd, *J* = 12.9, 9.3, 8.1 Hz, 1 H), 2.59 (ddd, 12.9, 6.1, 3.0 Hz). IR (neat): 2935, 1786, 1584, 1330, 1161, 1029, 746, 711, 589 cm^{-1}. HRMS (ESI): $[M+H]^+$ calculated for $C_{11}H_{11}ClO_2$: 211.0525. Found: 211.0526.

HPLC (ChiralPak IC, 20% *i*PrOH in hexanes, 1 mL/min, 254 nm)

racemic

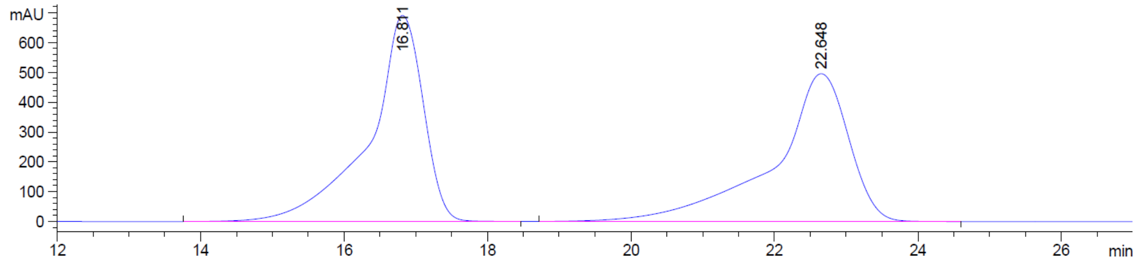

enantioenriched

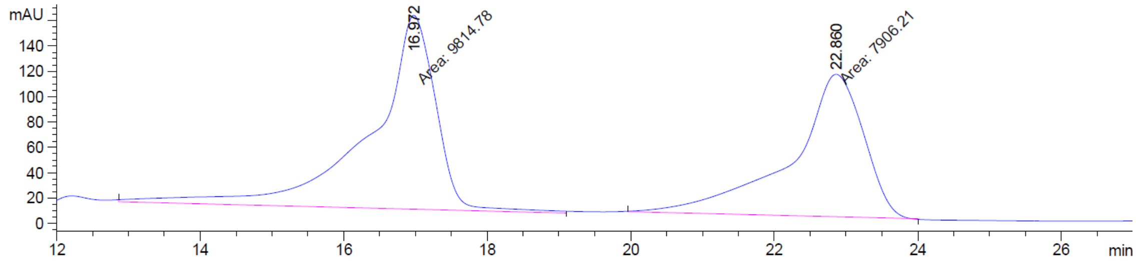

Peak	Retention time (min)	Area (mAU*s)	Area %
1	16.89	9814.8	55.38
2	22.75	7906.2	44.62

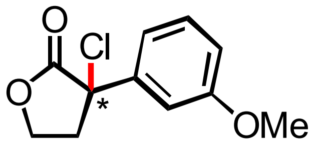

2g

3-Chloro-3-(3-methoxyphenyl)dihydrofuran-2(3*H*)-one (2g)

Substrate **1g** was chlorinated following the general procedure. **2g** was obtained as a colorless oil (38 mg, 84% yield, 80% ee). $[\alpha]^{25}_{D}$ = –1.9° (c = 1.0, $CHCl_3$). ^{1}H NMR (600 MHz, $CDCl_3$): δ = 7.33 (t, J = 8.0 Hz, 1 H), 7.26 (dd, J = 4.7, 2.5 Hz, 1 H), 7.20 (ddd, J = 7.8, 1.8, 0.8 Hz, 1 H), 6.92 (ddd, J = 8.2, 2.5, 0.8 Hz, 1 H), 4.57 (td, J = 8.9, 6.0 Hz, 1 H), 4.42 (ddd, J = 9.0, 7.3, 3.0 Hz, 1 H), 3.83 (s, 3 H), 3.00 – 2.90 (m, 2 H). ^{13}C NMR (126 MHz, $CDCl_3$): δ = 172.3, 159.8, 138.0, 129.8, 118.9, 114.7, 113.0, 65.4, 65.3, 55.4, 40.8. IR (neat): 2990, 1790, 1369, 1142, 1060, 1011, 841, 641, 599 cm^{-1}. HRMS (ESI): $[M+H]^+$ calculated for $C_{11}H_{11}ClO_3$: 227.0475. Found: 227.0477.

HPLC (ChiralPak IC, 20% *i*PrOH in hexanes, 1 mL/min, 254 nm)

racemic

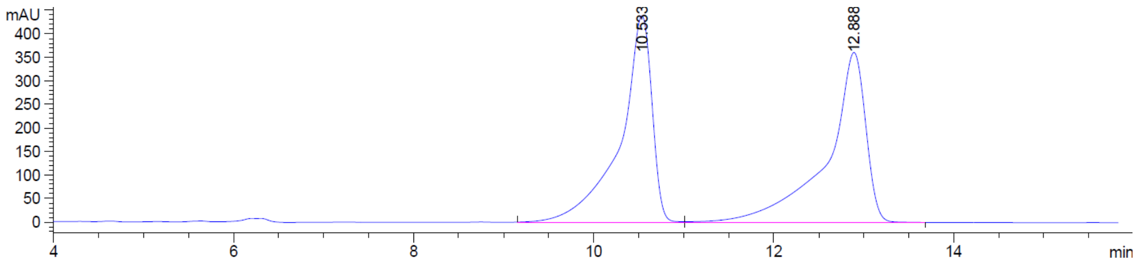

enantioenriched

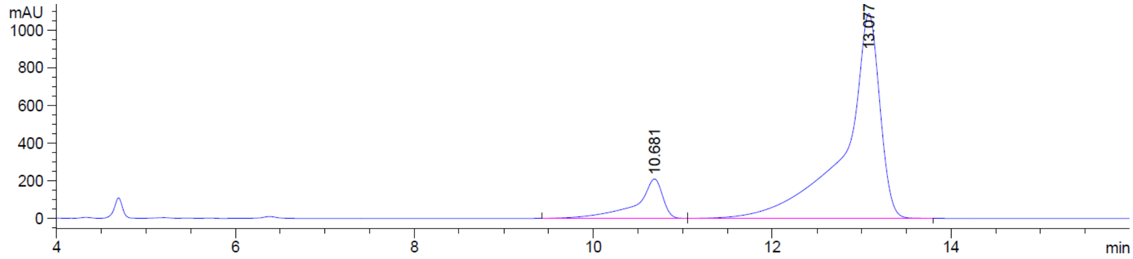

Peak	Retention time (min)	Area (mAU*s)	Area %
1	10.61	3408.4	10.21
2	12.94	29786	89.79

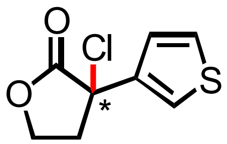

2h

3-Chloro-3-(thiophen-3-yl)dihydrofuran-2(3*H*)-one (2h)

Substrate **1h** was chlorinated following general procedure **B**. After purification by column chromatography, **2h** was obtained as a colorless oil (35 mg, 88% yield, 58% ee). $[\alpha]^{25}_{D}$ = –2.2° (c = 1.0, $CHCl_3$). ^{1}H NMR (600 MHz, $CDCl_3$): δ = 7.60 (dd, J = 3.0, 1.4 Hz, 1 H), 7.37 (dt, J = 4.9, 2.3 Hz, 1 H), 7.34 (m, 1 H), 4.56 (td, J = 8.8, 6.3 Hz, 1 H), 4.45 – 4.42 (m, 1 H), 2.99 – 2.91 (m, 2 H). ^{13}C NMR (126 MHz, $CDCl_3$): δ = 174.8, 140.0, 129.8, 129.2, 127.1, 68.1, 64.7, 42.7. IR (neat): 2968, 1748, 1465, 1136, 1121, 972, 901, 686, 600 cm^{-1}. HRMS (ESI): $[M+Na]^+$ calculated for $C_8H_7ClO_2S$: 224.9753. Found: 224.9756.

HPLC (ChiralPak IC, 10% *i*PrOH in hexanes, 1 mL/min, 254 nm)

racemic

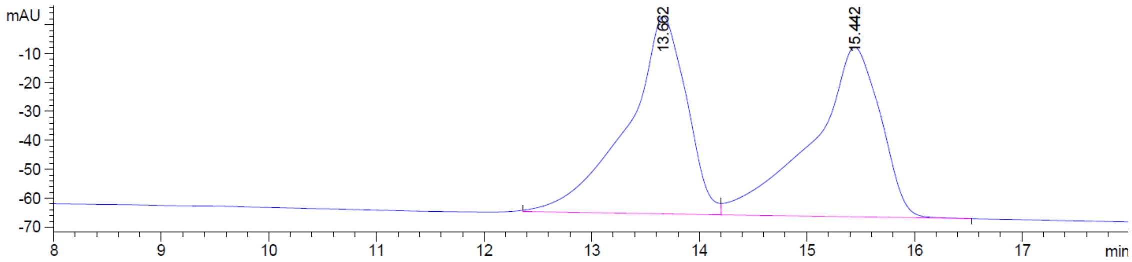

enantioenriched

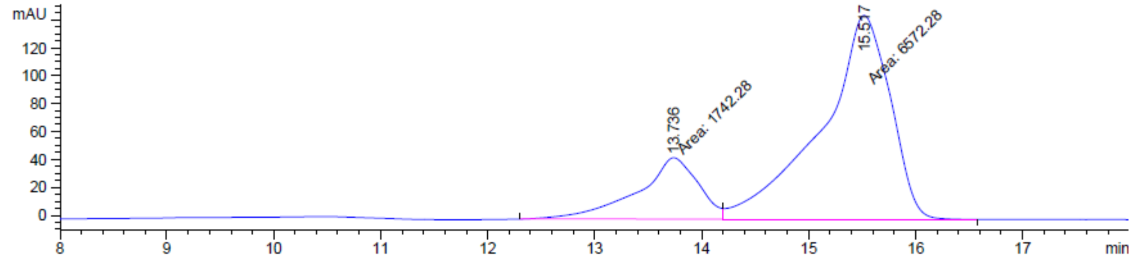

Peak	Retention time (min)	Area (mAU*s)	Area %
1	13.70	1472.3	20.95
2	15.48	6572.3	79.05

D. Substitution Reactions of α-Chloro Esters

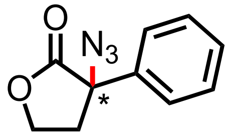

6

3-Azido-3-phenyldihydrofuran-2(3H)-one (6)

In a 20 mL scintillation vial, sodium azide (65 mg, 1.0 mmol) was added to a stirring solution of **2a** (98 mg, 0.5 mmol, 90% ee) in dry acetonitrile (4 mL).[5] After 4 hours at room temperature, the solvent was thoroughly removed, and the crude mixture was suspended in ethyl acetate (10 mL) and filtered to remove inorganic salts. Purification by column chromatography on silica, eluting with 30% EtOAc in hexanes, provided the desired product **6** as a colorless oil (92 mg, 91% yield, 85% ee). $[\alpha]^{25}_{D}$ = 1.1° (c = 1.0, $CHCl_3$). ^{1}H NMR (600 MHz, $CDCl_3$): δ = 7.50 – 7.41 (m, 5 H), 4.40 (ddd, *J* = 9.2, 7.0, 5.5 Hz, 1 H), 4.16 (ddd, *J* = 9.2, 7.6, 6.9 Hz, 1 H), 2.59 – 2.54 (m, 2 H). ^{13}C NMR (125 MHz, $CDCl_3$): δ = 171.9, 132.7, 127.0, 126.9, 124.1, 65.4, 62.8, 35.1. IR (neat): 2977, 1780, 1493, 1448, 1371, 1214, 1163, 1095, 950, 896, 834, 694, 641, 609 cm^{-1}. HRMS (ESI-TOF): $[M+Na]^+$ calculated for $C_{10}H_9N_3O_2$: 226.0593. Found: 226.0601.

HPLC (ChiralPak IC, 20% *i*PrOH in hexanes, 1 mL/min, 254 nm)

racemic

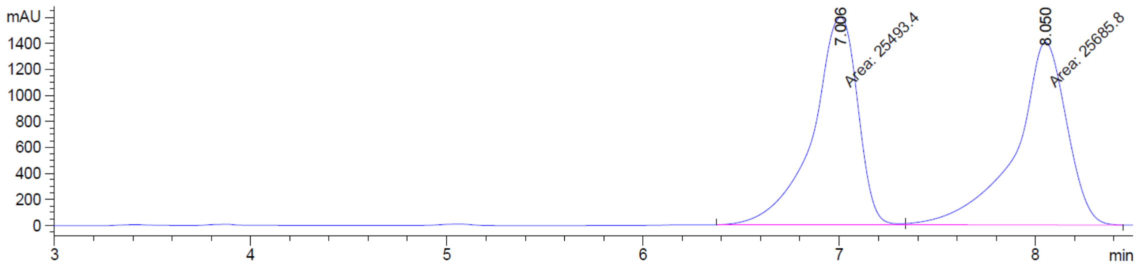

enantioenriched

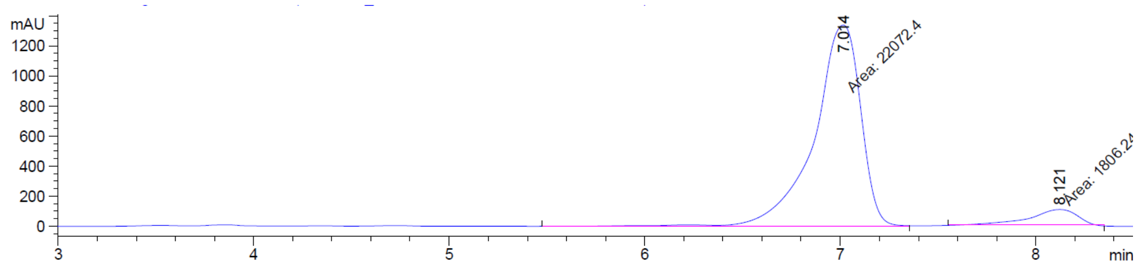

Peak	Retention time (min)	Area (mAU*s)	Area %
1	7.01	22072	92.44
2	8.09	1806.2	7.56

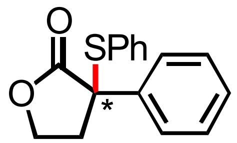

7

3-Phenyl-3-(phenylthio)dihydrofuran-2(3H)-one (7)

In a 1 dram vial, thiophenol sodium salt (133 mg, 1.0 mmol) was added to a stirring solution of **2a** (98 mg, 0.5 mmol, 90% ee) in dry acetonitrile (4 mL).[5] The vial was sealed tightly and submerged partially in an oil bath at 90 °C. After stirring overnight, the reaction was cooled to room temperature and water (3 mL) was added. The mixture was extracted with dichloromethane (3 x 5 mL) and concentrated. Purification by column chromatography on silica, eluting with 30% EtOAc in hexanes, provided the desired product **7** as a slightly yellow viscous oil (89 mg, 66% yield, 86% ee). $[\alpha]^{25}_{D}$ = 2.5° (c = 1.0, $CHCl_3$). ^1H NMR (600 MHz, $CDCl_3$): δ = 7.55 (t, *J* = 7.4 Hz, 1 H), 7.48 – 7.46 (m, 2 H), 7.39 – 7.36 (m, 2 H), 7.34 – 7.30 (m, 3 H), 7.26 – 7.23 (m, 2 H), 4.66 – 4.62 (m, 1 H), 4.31 (td, *J* = 8.4, 5.2 Hz, 1 H), 3.73 (ddd, *J* = 14.1, 7.9, 5.2 Hz, 1 H), 3.01 (ddd, *J* = 14.1, 8.1, 7.0 Hz, 1 H). ^{13}C NMR (125 MHz, $CDCl_3$): δ = 170.0, 144.2, 134.3, 133.7, 131.3, 131.0, 129.5, 129.3, 128.5, 128.4, 128.0, 127.0, 124.9, 123.7, 74.1, 65.5, 31.9. IR (neat): 2980, 1710, 1449, 1225, 1166, 1020, 953, 849, 767, 690 cm^{-1}. HRMS (ESI-TOF): $[M+H]^+$ calculated for $C_{16}H_{14}O_2S$: 293.0612. Found: 293.0612.

HPLC (ChiralPak IC, 15% *i*PrOH in hexanes, 1 mL/min, 254 nm)

racemic

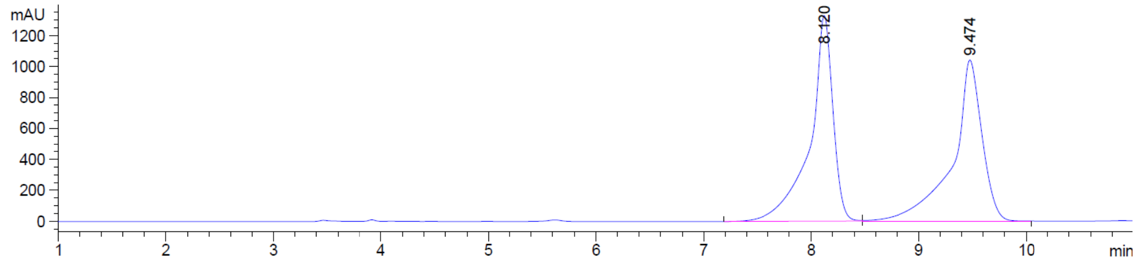

enantioenriched

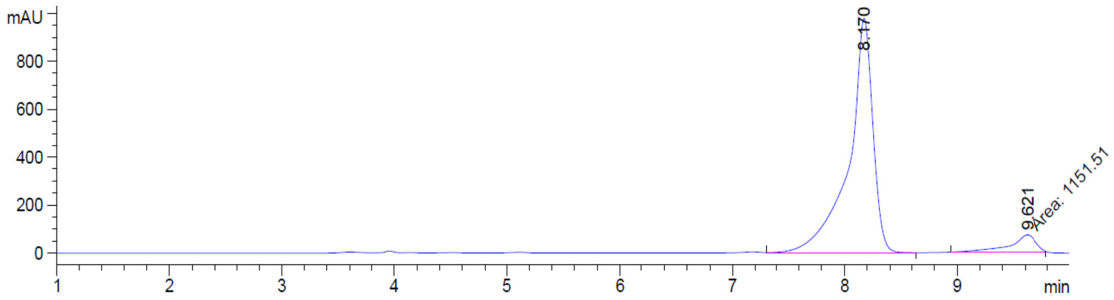

Peak	Retention time (min)	Area (mAU*s)	Area %
1	8.15	15431	93.06
2	9.55	1151.5	6.94

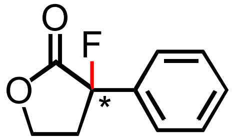

8

3-Fluoro-3-phenyldihydrofuran-2(3H)-one (8)

In a flame-dried 1 dram vial, equipped with a magnetic stir bar and a rubber septum cap, cesium fluoride (45.6 mg, 0.3 mmol, anhydrous) and 18-crown-6 (26.4 mg, 0.1 mmol) was added quickly to a stirring solution of **2a** (19.7 mg, 0.1 mmol) in *tert*-butanol (0.5 mL).[5] The vial was sealed tightly and submerged partially in an oil bath at 60 °C with vigorous stirring. 5 hours later, the reaction was cooled to room temperature and diluted with dichloromethane (10 mL). After filtration and concentration, the crude material was subjected to column chromatography on silica, eluting with 25% EtOAc in hexanes, providing the desired product **8** as a clear, viscous oil (4.5 mg, 25% yield, 89% ee). $[\alpha]^{25}_{D}$ = 2.1° (c = 1.0, $CHCl_3$). ^1H NMR (600 MHz, $CDCl_3$): δ = 7.49 – 7.42 (m, 5 H), 4.55 (m, 1 H), 4.37 (m, 1 H), 2.93 – 2.78 (m, 2 H). ^{13}C NMR (125 MHz, $CDCl_3$): δ = 172.2 (d, J = 25.2 Hz), 134.9 (d, J = 23.1 Hz), 129.7 (d, J = 2.5 Hz), 128.9, 125.2 (d, J = 6.8 Hz), 93.9 (d, J = 188.2 Hz), 64.9 (d, J = 3.9 Hz), 36.3 (d, J = 23.9 Hz). ^{19}F NMR (376 MHz, $CDCl_3$): δ = –150.8 (t, J = 20.3 Hz). IR (neat): 2977, 1771, 1612, 1361, 1253, 1144, 1093, 1024, 844, 659 cm^{-1}. HRMS (ESI-TOF): $[M+Na]^+$ calculated for $C_{10}H_9FO_2$: 203.0484. Found: 203.0490.

HPLC (ChiralPak IC, 10% *i*PrOH in hexanes, 1 mL/min, 254 nm)

racemic

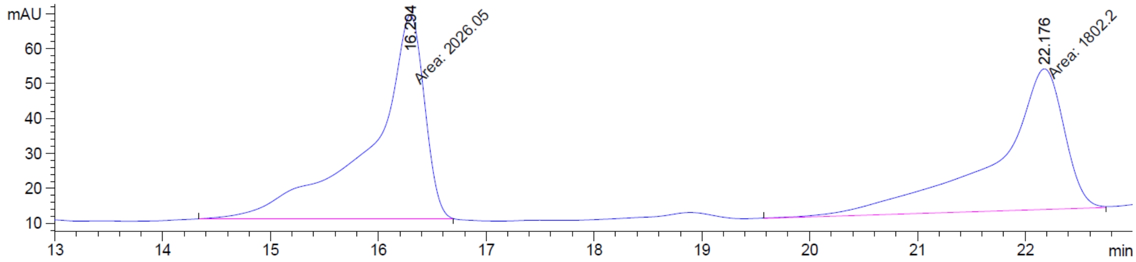

enantioenriched

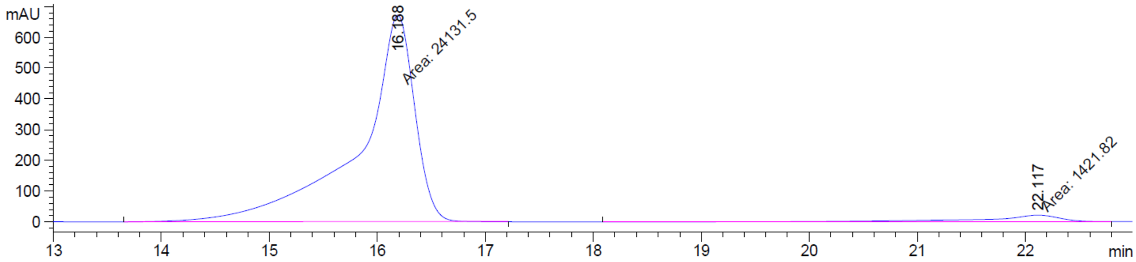

Peak	Retention time (min)	Area (mAU*s)	Area %
1	16.24	24132	94.44
2	22.15	1421.8	5.56

References

1. **S1** was synthesized according to previously reported procedure: Knowles, R. R.; Lin, S.; Jacobsen, E. N. *J. Am. Chem. Soc.* **2010**, *132*, 5030.
2. **S2** was synthesized according to previously reported procedure: Yang, W.; Du, D.–M. *Org. Lett.* **2010**, *12*, 5450.
3. Thiourea and urea catalysts **3** and **4** were synthesized according to previously reported procedure: Lin, S.; Jacobsen, E. N. *Nat. Chem.* **2012**, *4*, 817.
4. Birrell, J. A.; Desrosiers, J.–N.; Jacobsen, E. N. *J. Am. Chem. Soc.* **2011**, *133*, 13872.
5. The procedures are based on those reported by: Shibatomi, K.; Soga, Y; Narayama, A.; Fujisawa, I.; Iwasa, S. *J. Am. Chem. Soc.*, **2012**, *134*, 9836.

NMR Spectra of Products

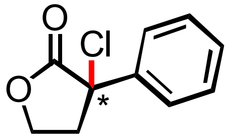

2a

^{1}H NMR ($CDCl_3$, 600 MHz)

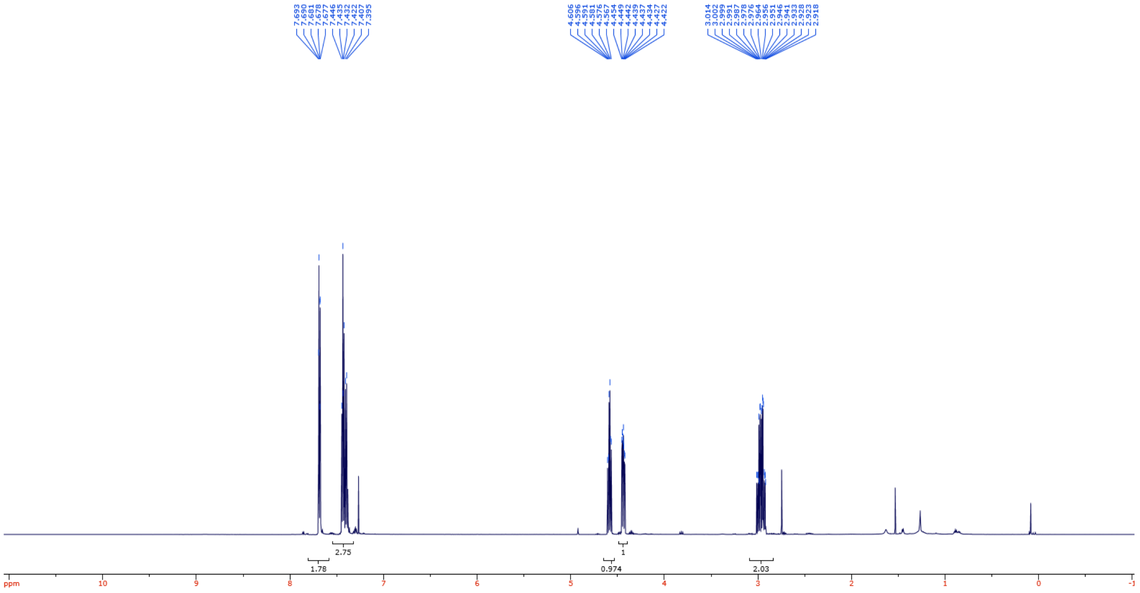

^{13}C NMR ($CDCl_3$, 126 MHz)

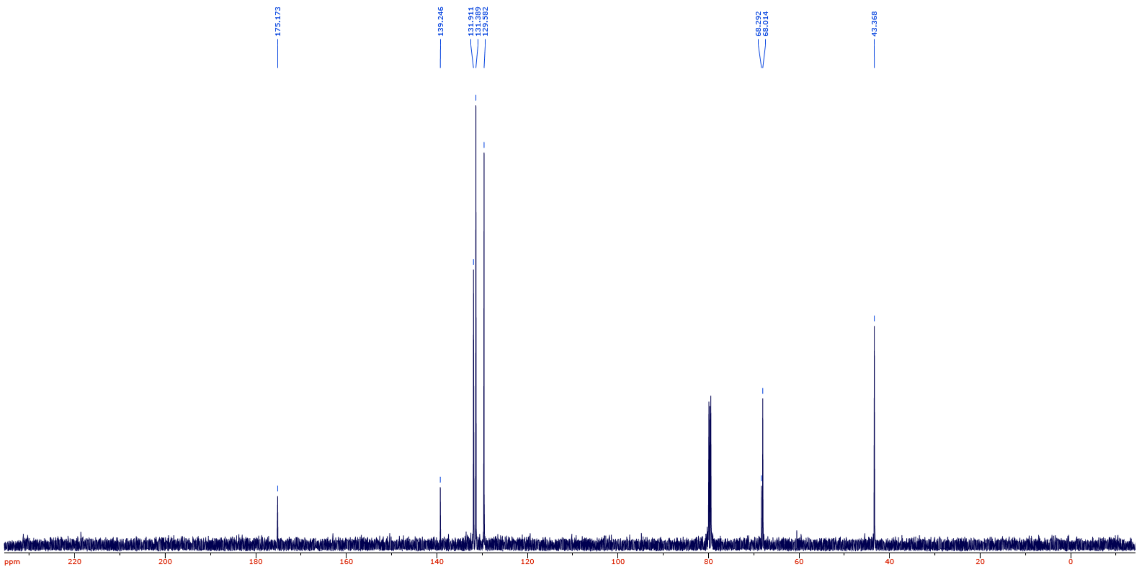

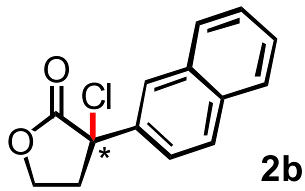

2b

^{1}H NMR ($CDCl_3$, 600 MHz)

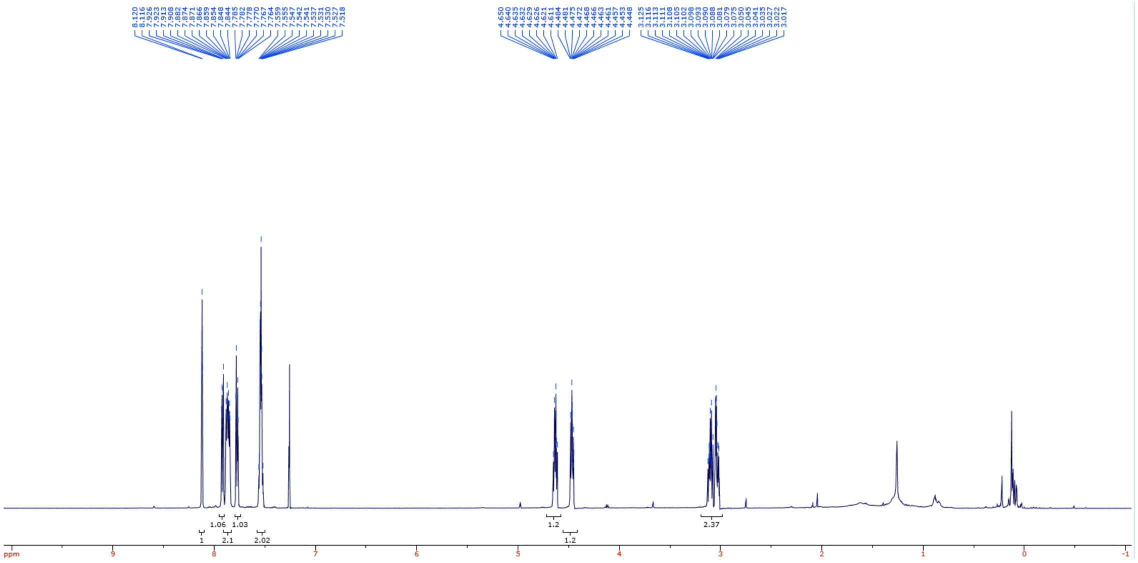

^{13}C NMR ($CDCl_3$, 126 MHz)

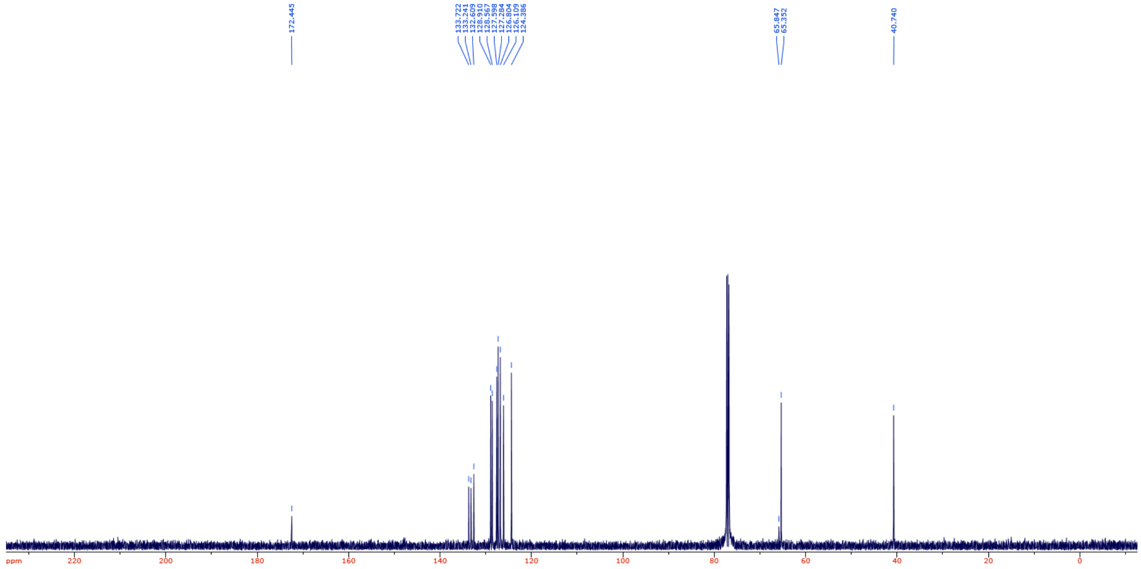

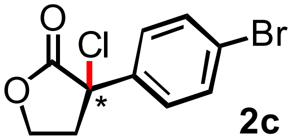

2c

^{1}H NMR ($CDCl_3$, 500 MHz)

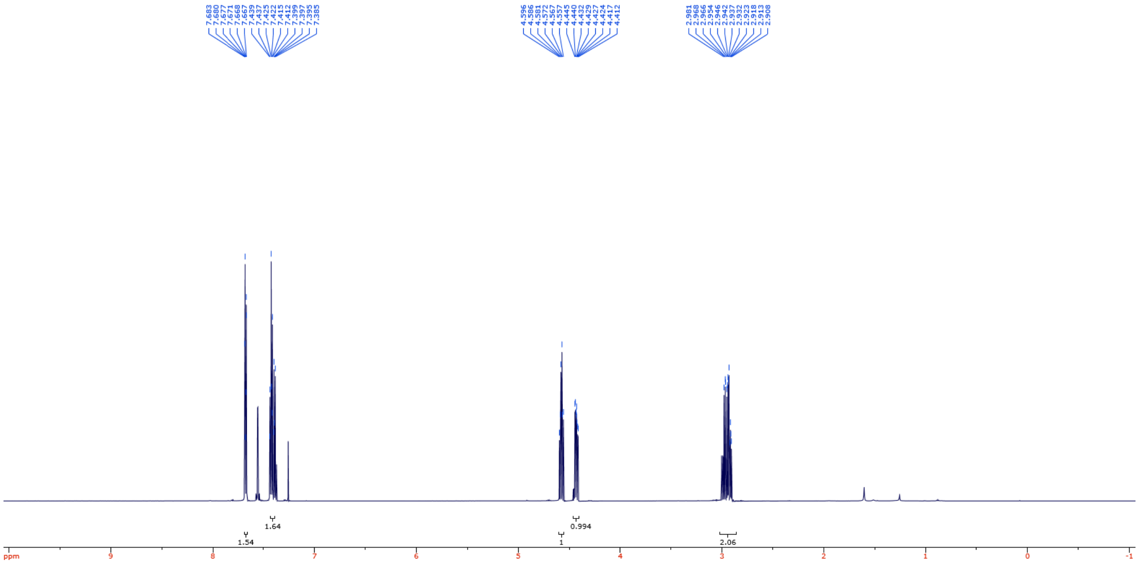

^{13}C NMR ($CDCl_3$, 126 MHz)

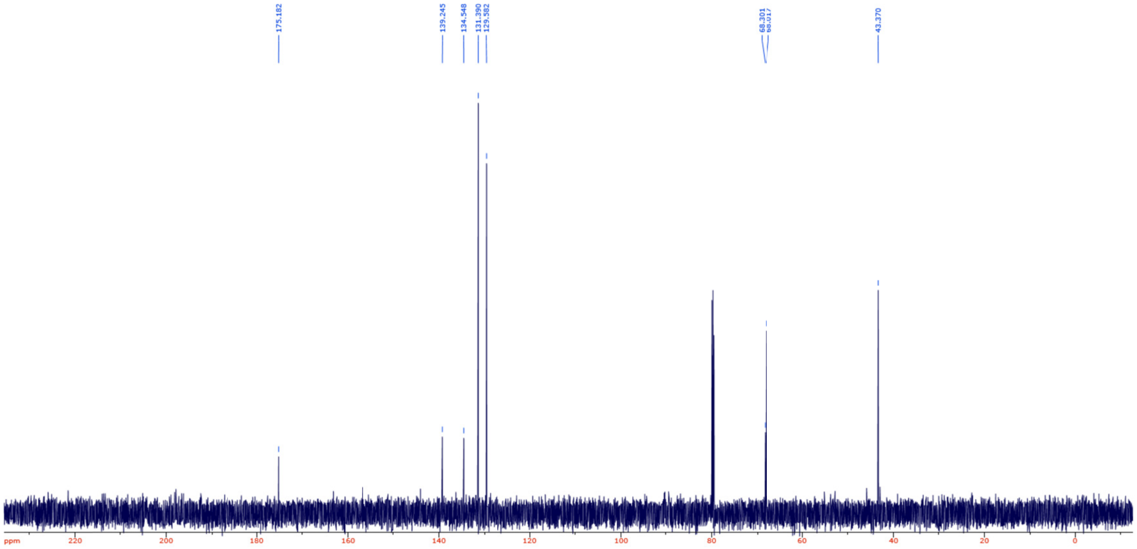

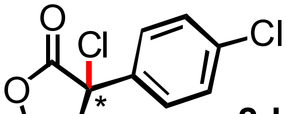

2d

^{1}H NMR ($CDCl_3$, 600 MHz)

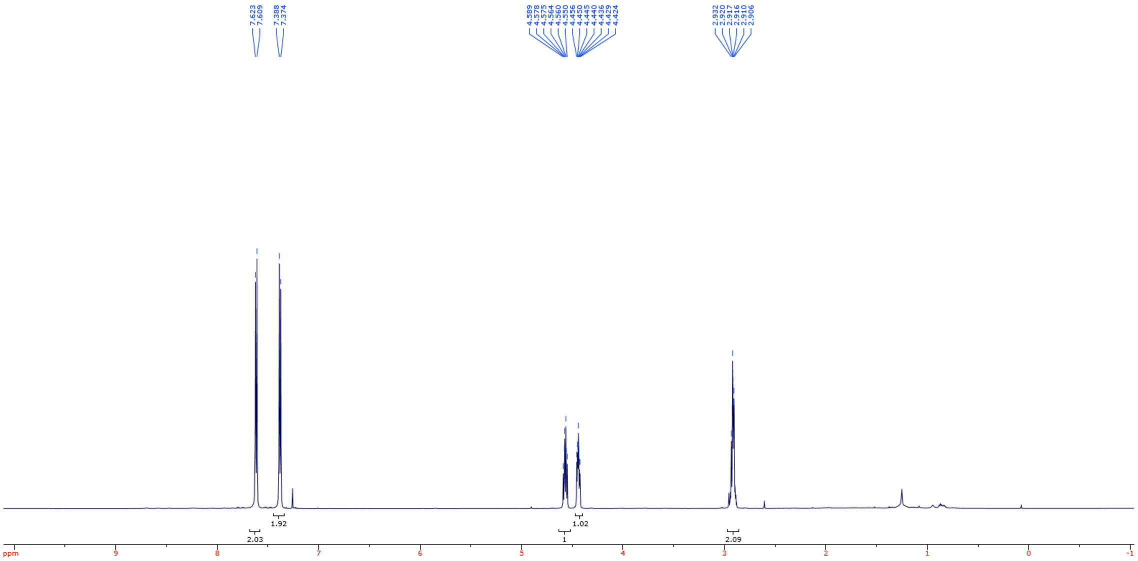

^{13}C NMR ($CDCl_3$, 126 MHz)

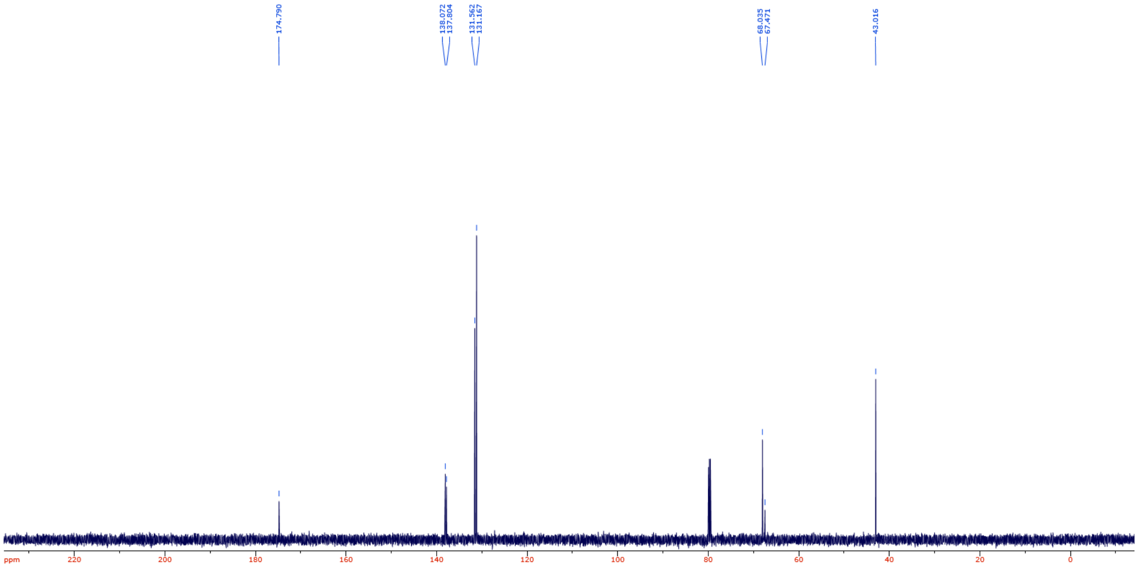

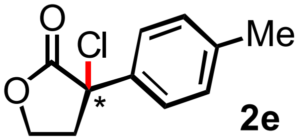

2e

^{1}H NMR ($CDCl_3$, 600 MHz)

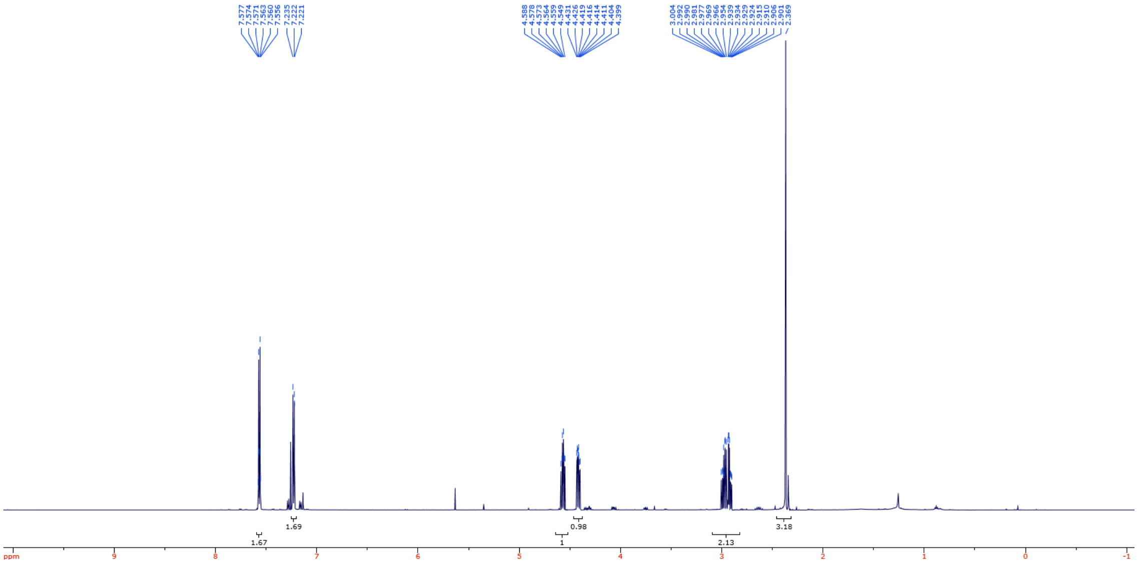

^{13}C NMR ($CDCl_3$, 126 MHz)

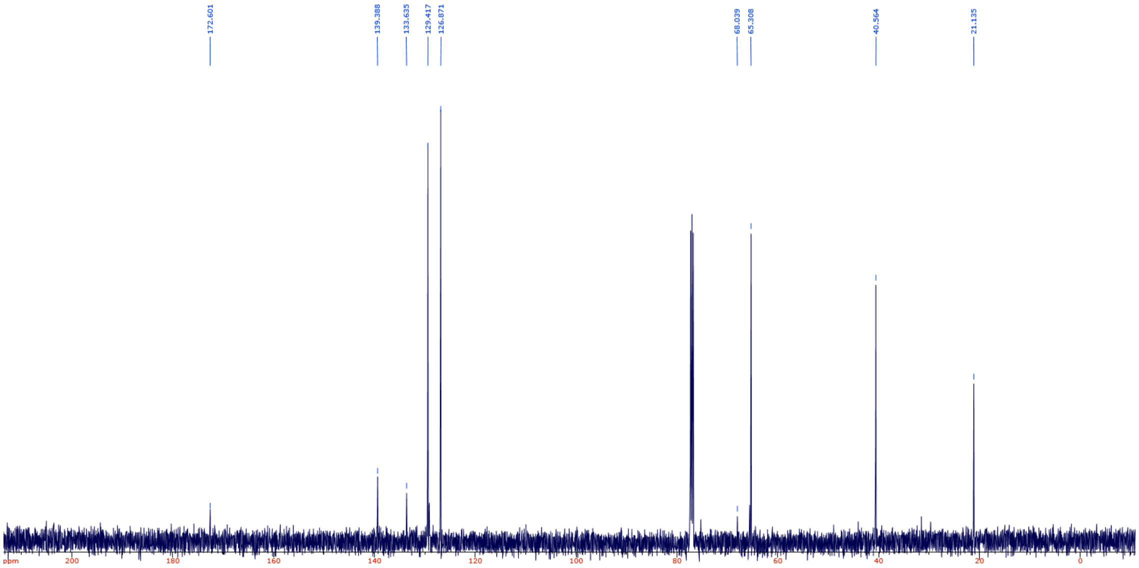

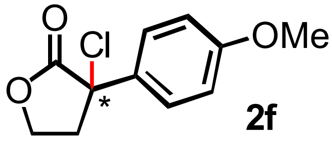

2f

^{1}H NMR ($CDCl_3$, 600 MHz)

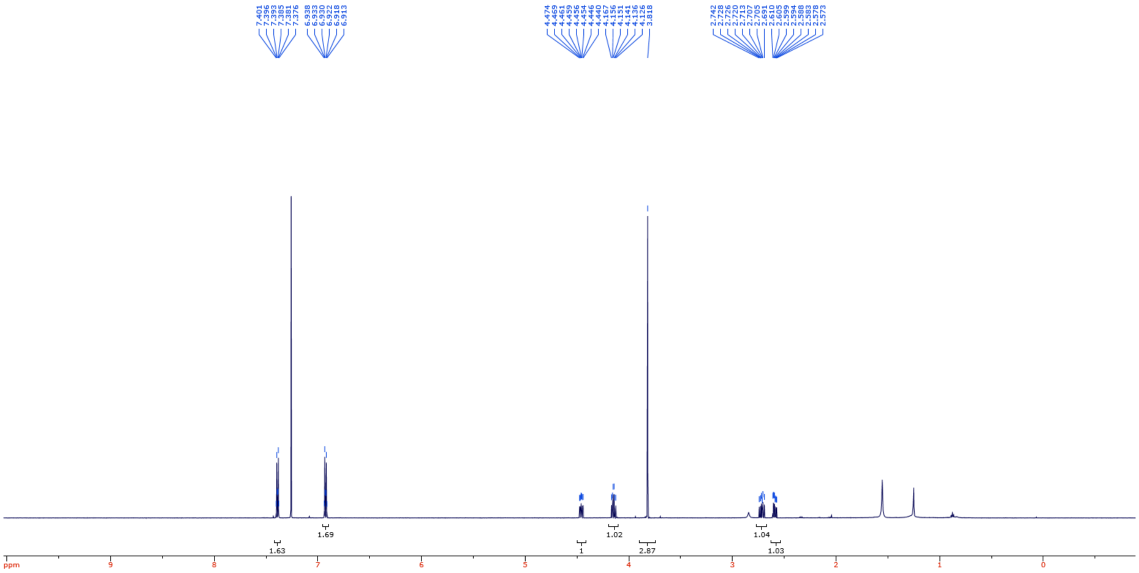

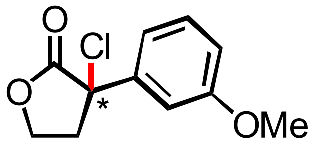

2g

^{1}H NMR ($CDCl_3$, 600 MHz)

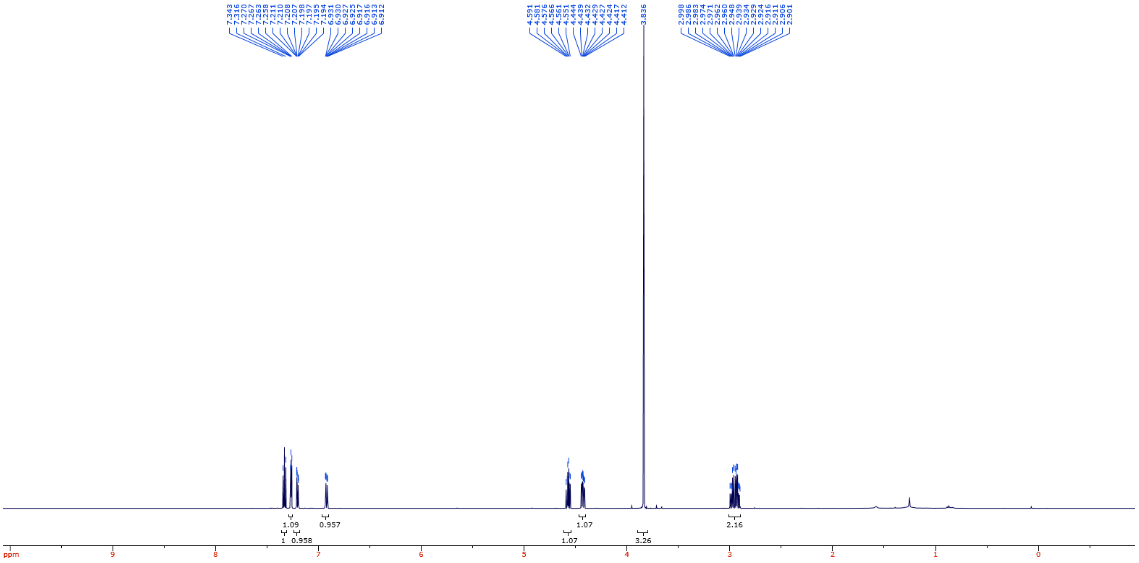

^{13}C NMR ($CDCl_3$, 126 MHz)

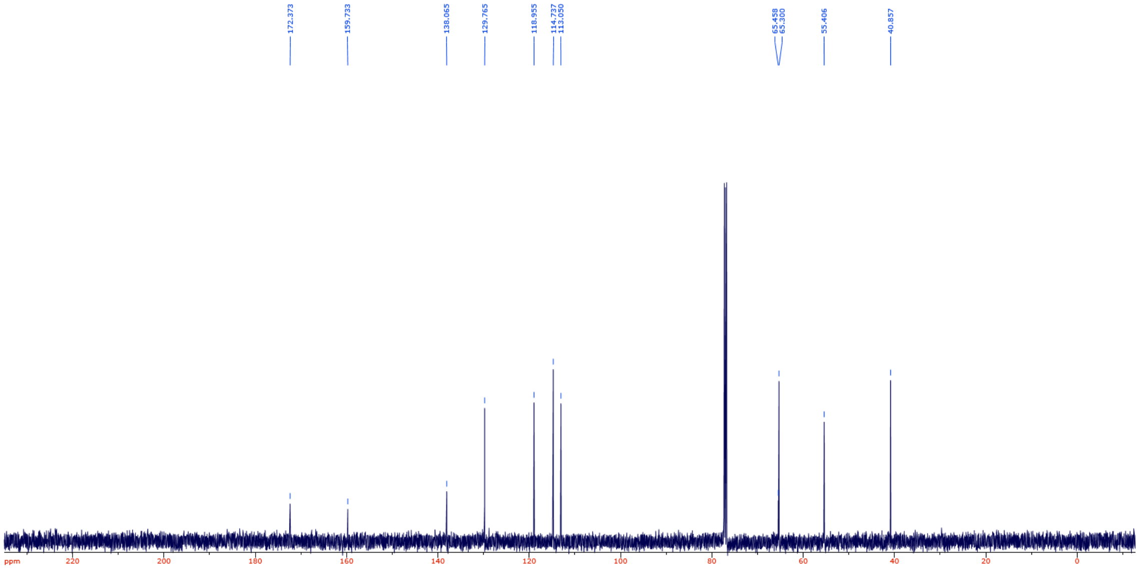

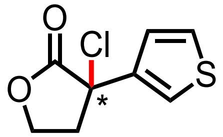

2h

^{1}H NMR (CDCl$_3$, 600 MHz)

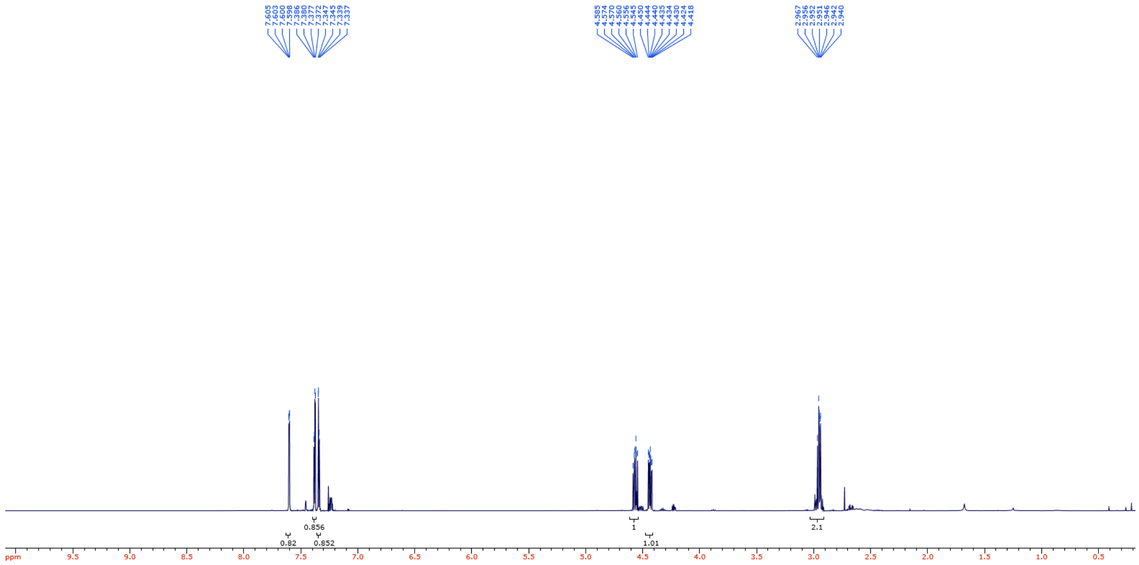

^{13}C NMR (CDCl$_3$, 126 MHz)

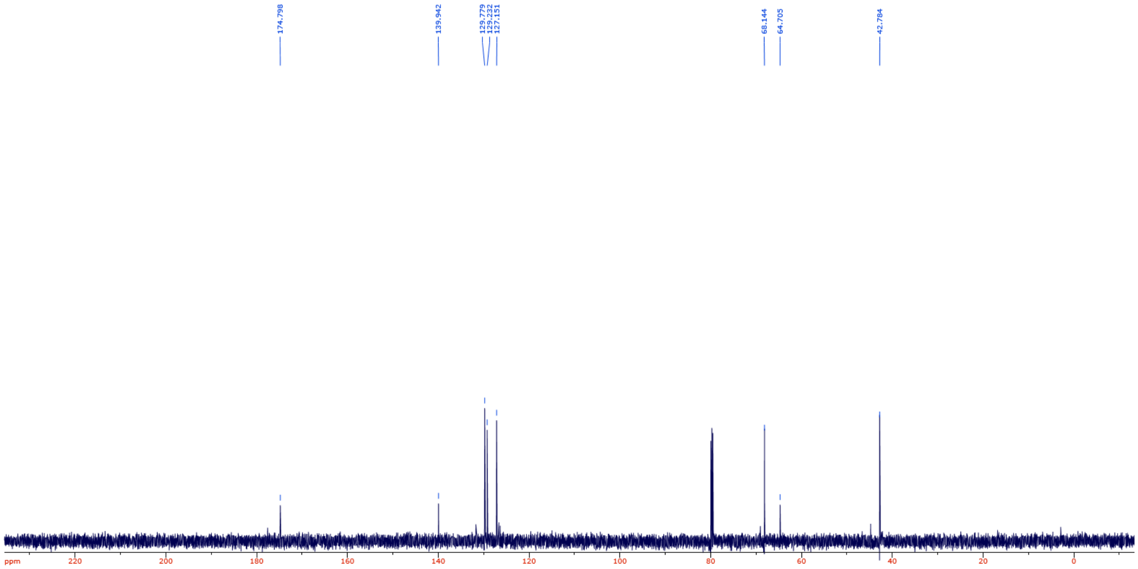

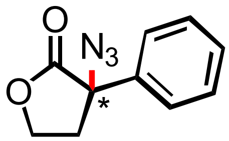

6

^{1}H NMR ($CDCl_3$, 600 MHz)

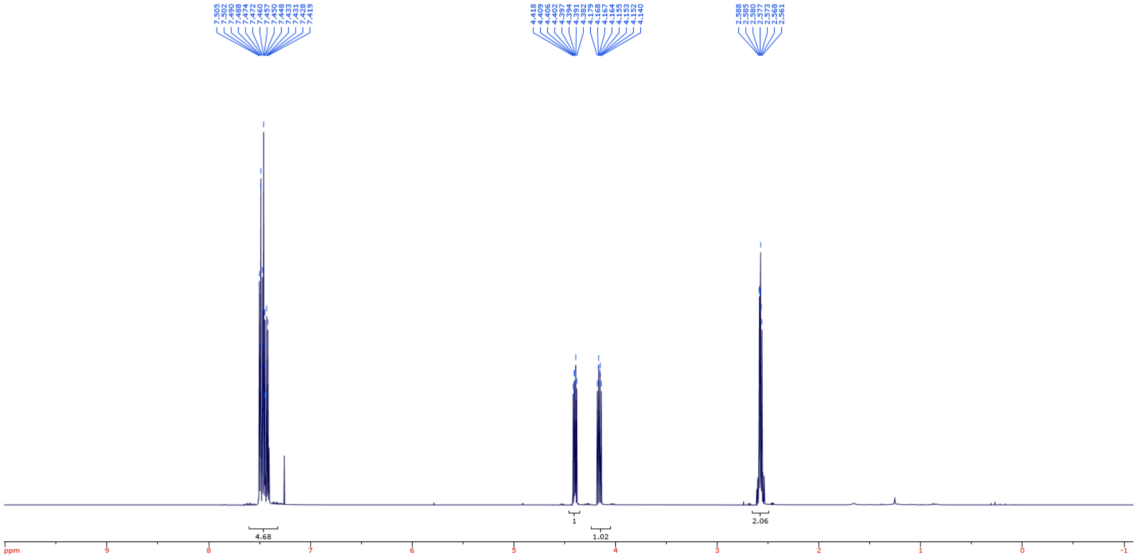

^{13}C NMR ($CDCl_3$, 126 MHz)

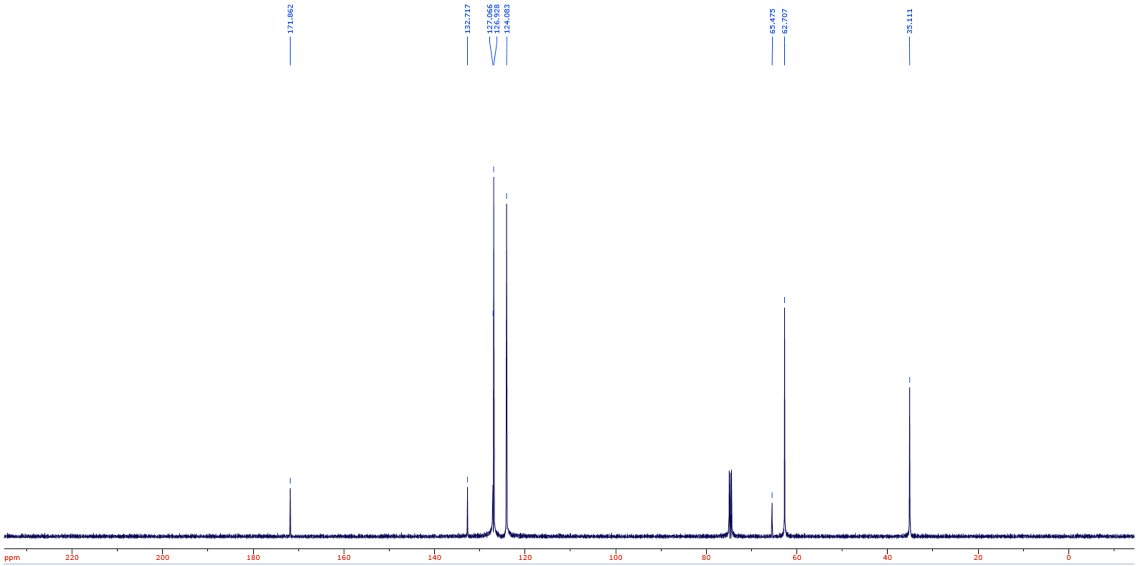

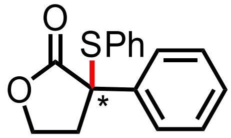

7

^{1}H NMR (CDCl$_3$, 600 MHz)

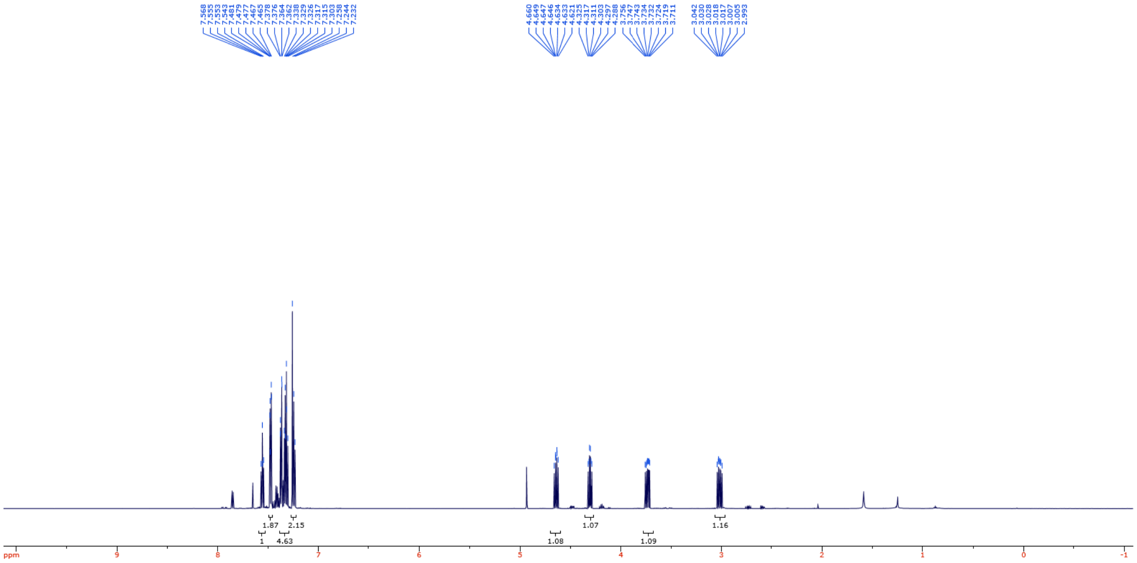

1

^{13}C NMR (CDCl$_3$, 126 MHz)

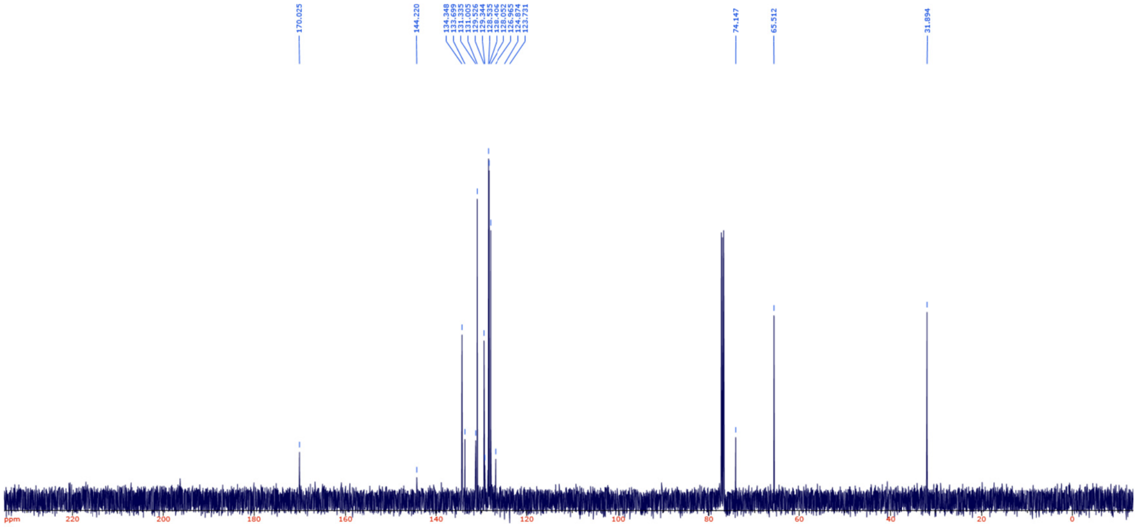

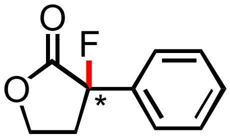

8

^{1}H NMR ($CDCl_3$, 600 MHz)

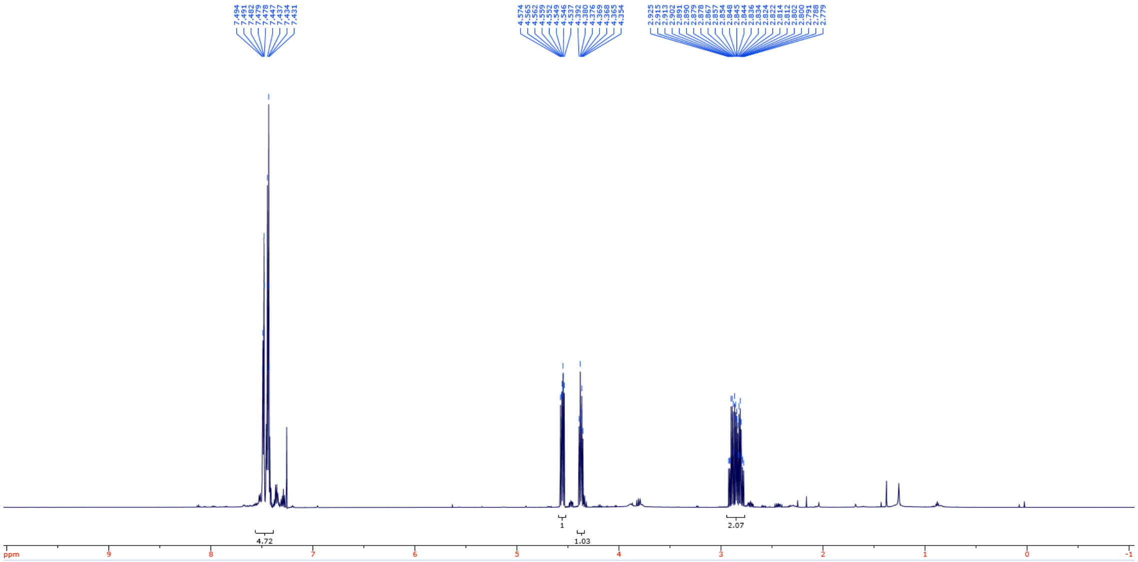

^{13}C NMR ($CDCl_3$, 126 MHz)

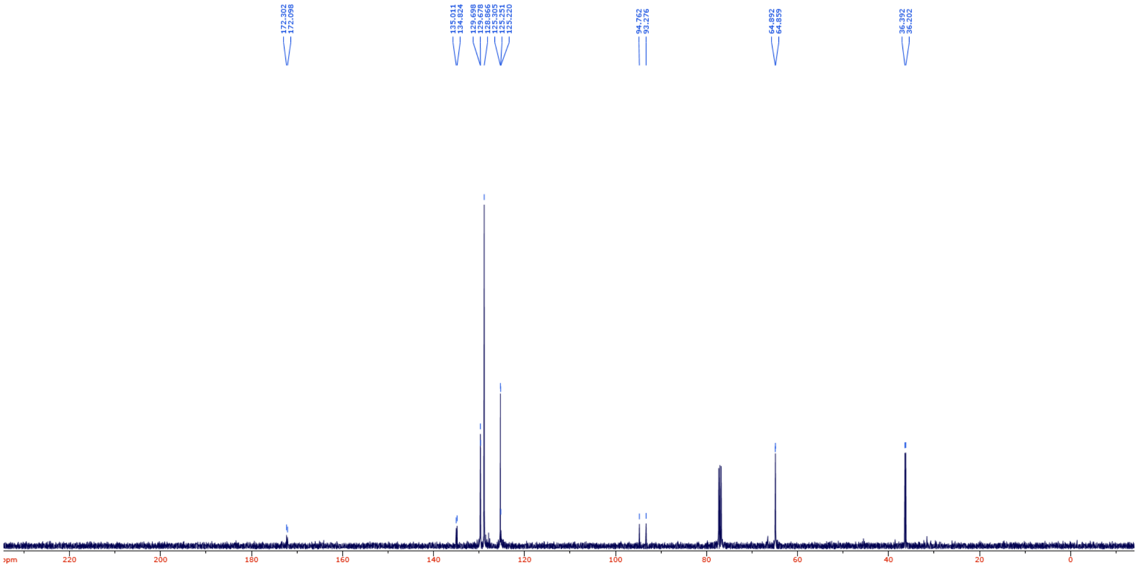

^{19}F NMR ($CDCl_3$, 375 MHz)

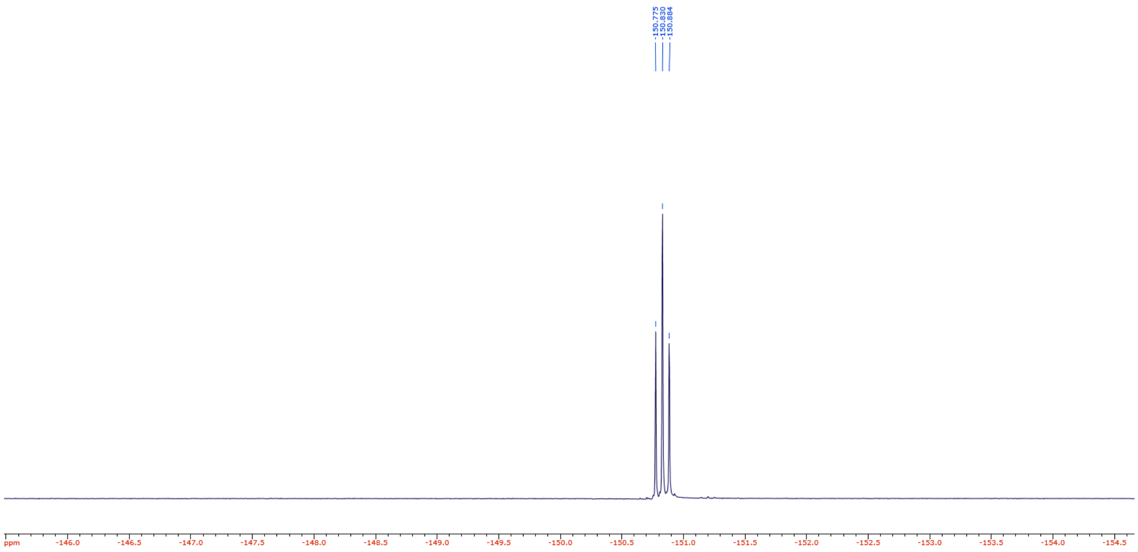

Supplement: SI [file NIHMS661548-supplement-SI.pdf]
